# Design, Synthesis, and Biological Evaluation of 1,2,3-Triazole-linked triazino[5,6-b]indole-benzene sulfonamide Conjugates as Potent Carbonic Anhydrase I, II, IX, and XIII Inhibitors

**DOI:** 10.3390/metabo10050200

**Published:** 2020-05-15

**Authors:** Krishna Kartheek Chinchilli, Andrea Angeli, Pavitra S. Thacker, Laxman Naik Korra, Rashmita Biswas, Mohammed Arifuddin, Claudiu T. Supuran

**Affiliations:** 1 Department of Medicinal Chemistry, National Institute of Pharmaceutical Education and Research (NIPER), Balanagar, Hyderabad 500037, India; krishnakrthk22@gmail.com (K.K.C.); thackerp42@yahoo.com (P.S.T.); laxmanaik827@gmail.com (L.N.K.); rashmitabiswas06@gmail.com (R.B.); 2 Neurofarba Department, Sezione di Scienze Farmaceutiche e Nutraceutiche, Università degli Studi di Firenze, Via Ugo Schiff 6, Sesto Fiorentino, 50019 Florence, Italy; andrea.angeli@unifi.it; 3 Department of Chemistry, Anwarul Uloom College, 11-3-918, New Malleypally, Hyderabad 500001, India

**Keywords:** 1,2,3-triazole, triazino[5,6-b]indole-benzene sulfonamide, carbonic anhydrase inhibitors

## Abstract

A series of 1,2,3-triazole-linked triazino[5,6-b]indole-benzene sulfonamide hybrids (**6a–6o**) was synthesized and evaluated for carbonic anhydrase (CA, EC 4.2.1.1) inhibitory activity against the human (h) isoforms hCA I, II, XIII (cytosolic isoforms), and hCA IX (transmembrane tumor-associated isoform). The results revealed that the compounds **6a–6o** exhibited K_i_ values in the low to medium nanomolar range against hCA II and hCA IX (K_i_s ranging from 7.7 nM to 41.3 nM) and higher K_i_ values against hCA I and hCA XIII. Compound **6i** showed potent inhibition of hCA II (K_i_ = 7.7nM), being more effective compared to the standard inhibitor acetazolamide (AAZ) (K_i_ = 12.1 nM). Compounds **6b** and **6d** showed moderate activity against hCA XIII (K_i_ = 69.8 and 65.8 nM). Hence, compound **6i** could be consider as potential lead candidate for the design of potent and selective hCA II inhibitors.

## 1. Introduction

Carbonic anhydrases (CAs, EC 4.2.1.1) are omnipresent metalloenzymes that they play a pivotal catalytic role in the hydration of carbon dioxide to bicarbonate and protons by means of a ping pong mechanism, which is a slow process under non-catalytic conditions [1,2,3,4].

$$CO_{2}+H_{2}O\underset{}{\Leftrightarrow }HCO_{3}{}^{-}+H^{+}$$

These enzymes are encoded by eight genetically unrelated gene families, namely, α, β, γ, δ, ζ, η, θ, and the recently reported ι class [5]. Among these, the α family is predominantly present in mammals, and 16 isoforms have been reported with different catalytic activity, subcellular localization, and tissue distribution [1,2,3,4]. There are five cytosolic forms (CA I, CA II, CA III, CA VII, and CA XIII), five membrane-bound isozymes (CA IV, CA IX, CA XII, CA XIV, and CA XV), two mitochondrial forms (CA VA and CA VB), and a secreted isozyme (CA VI) [1,2,3,4]. These isoforms are implicated in different diseases, as shown in Table 1. Therefore, selective inhibition of a particular isoform redresses the particular disease in which it plays a major role.

**Figure 1:**
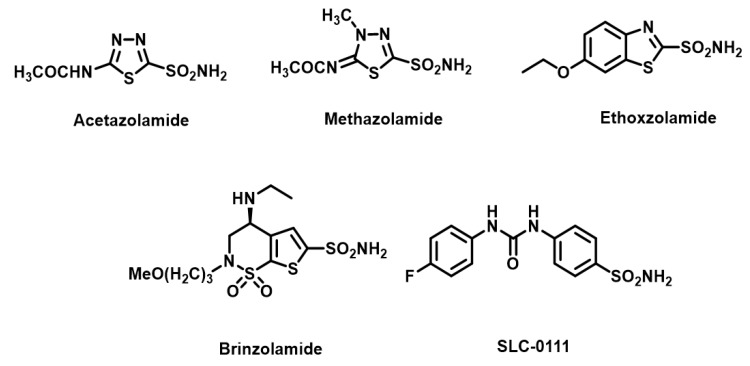
Structures of some clinically used sulfonamides.

To date, the sulfonamide group is considered as main zinc-binding group for the design of carbonic anhydrase inhibitors. Sulfonamides and their bio-isosteres such as sulfamides / sulfamates are known to elicit potent carbonic anhydrase inhibition and hence they are present in drugs, which are prescribed for the treatment of glaucoma, epilepsy, obesity, and cancer. The diuretic drugs mainly target CA II, CA IV, CA XII, and CA XIV [6,7], the anti-glaucoma drugs target CA II, CA IV, and CA XII [8,9], while the anti-epileptics target CA VII and CAXIV [10,11,12]. CA IX and CA XII specifically expressed in tumor cells, and their inhibition results in anti-metastatic effects [13,14,15]. However, the main drawback of all these drugs is the lack of selectivity, which results in serious side effects. Therefore, there is an urgent need to design and develop selective isoform inhibitors. The tail approach has been very successful in addressing this issue, and many novel scaffolds have been developed [16,17]. In this approach, the attached tails bind to the active site cavity, preferably the middle and the rim part, which shows variation in different CA isoforms. Some clinically/pre-clinically used sulfonamides are illustrated in the Figure 1:

Owing to the development of novel carbonic anhydrase inhibitors with better isoform selectivity, our group designed some novel hybrids in which the triazino[5,6-b]indole tail was conjugated to benzene sulfonamide via a 1,2,3-triazole linker. Triazino[5,6-b]indole is a flexible tail with diverse pharmacological activities, like anti-fungal/anti-bacterial [18], anti-diabetic [19], anti-depressant [20], anti-hypertensive [21], anti-inflammatory [22], and anti-hypoxic activities [23].

**Figure 2:**
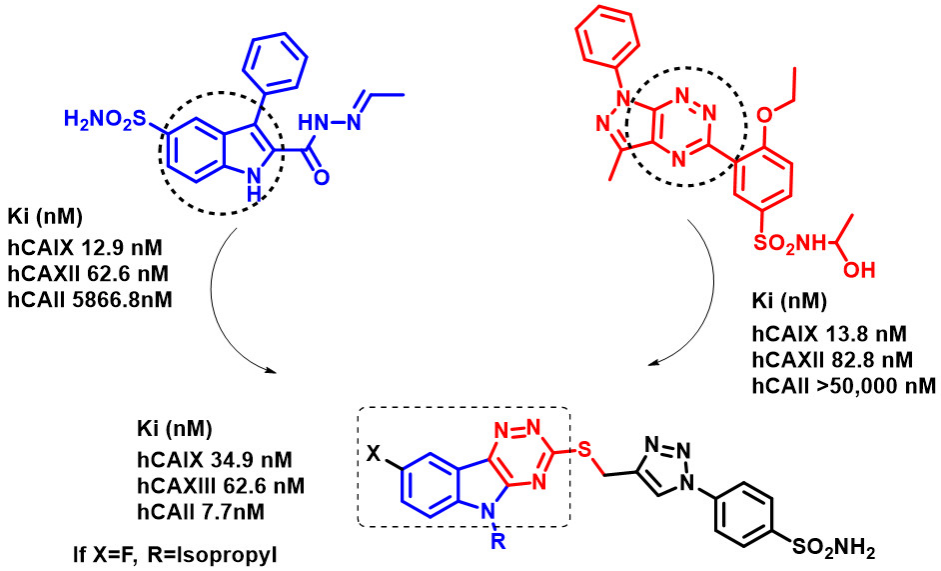
Design of target molecules by a molecular hybridization approach for hCA: human carbonic anhydrase.

The design of this new series of compounds was based on the tail approach via the fusion of indole and 1,2,4-triazine, which were reported for high interactions with carbonic anhydrase (Figure 2) [24,25,26,27,28]. The present design mainly is mainly involved two strategies. The first one was to fuse the two CA-binding scaffolds i.e., Indole and 1,2,4-triazine in order to develop a flexible tail with better interactions in the enzymatic site and the second one was to incorporate different N-alkyl substituents in the indole tail in a systematic fashion to define optimal length (methyl, ethyl, propyl), bulkiness (isopropyl) and un-saturation (allyl), which would confer the best CA inhibitory activity. It is reported in the literature that 1,2,3-triazole is an efficient linker, useful in the design of potent CA inhibitors, as it is an amide bioisostere and maintains high stability under basic as well as acidic hydrolysis conditions. It also has high dipole moment and capability of H bonding in vivo. Due to its aromatic character, it shows some π-stacking interactions with relevant amino acid residues [29].

## 2. Results and Discussion

### 2.1. Synthesis of the Target Molecules

The current design of experiment (DOE) was based on the molecular hybridization approach. We synthesized molecular hybrids of a bulky triazino[5,6-b]indole, used as a tail, conjugated to benzene sulfonamide through a flexible 1,2,3-triazole linker.

**Scheme 1:**
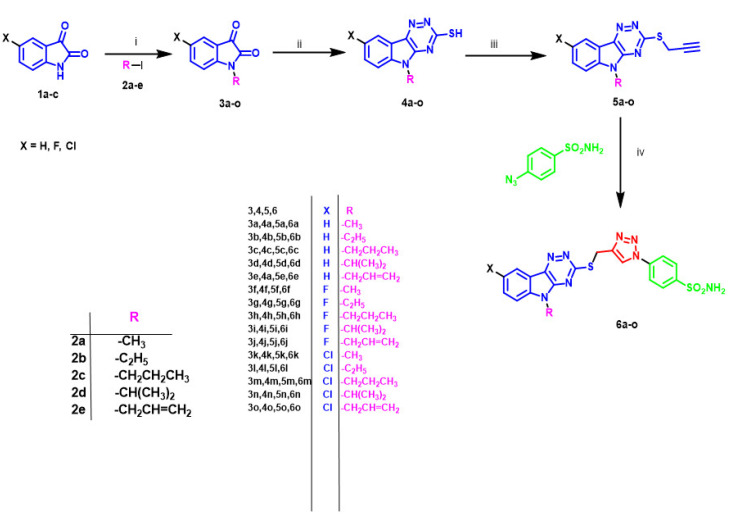
Synthesis of target 1,2,3-triazole-linked triazino[5,6-b]indole-benzene sulfonamide hybrids (**6a–6o**). Reagents and conditions: (i) K_2_CO_3_, KI (0.05 mole%), DMF, reflux, 4–6 h; yield: 72–75%; (ii) thiosemicarbazide, Cs_2_CO_3_, 1,4-dioxane, reflux, overnight; yield: 68–70%; (iii) propargyl bromide, K_2_CO_3_, DMF, rt, overnight; yield: 86–90%; (iv) CuSO_4_·5H_2_O, sodium ascorbate, tBuOH:H_2_O (1:1), 60 °C, overnight; yield: 65–70%.

The synthesis of 1,2,3-triazole-linked triazino[5,6-b]indole-benzene sulfonamide hybrids (**6a–6o**) was performed according to the general synthetic scheme illustrated in Scheme 1. The intermediate compounds (**4a–o**) were synthesized according to previously reported methods [30,31]. The *N*-alkylated isatins (**3a–o**) were synthesized from the simple five-substituted isatins (**1a–c**) by nucleophilic substitution of different alkyl halides (**2a–e**). The **3a–o** were condensed with thiosemicarbazide in aqueous 1,4-dioxane under reflux conditions by using cesium carbonate as a base followed by propargylation to generate intermediates (**5a–o**). Finally the **5a–o** were subjected to click reaction with 4-azido benzene sulfonamide to generate 1,2,3-triazole-linked triazino[5,6-b]indole-benzene sulfonamide hybrids (**6a–o**).

### 2.2. Carbonic Anhydrase Inhibition

The newly synthesized 1,2,3-triazole linked triazino[5,6-b]indole-benzene sulfonamide hybrids (**6a–6o**) were evaluated for their carbonic anhydrase inhibitory activity against a panel of carbonic anhydrases, i.e., hCA I, hCA II, hCA IX, and hCA XIII, by the stopped-flow CO_2_ hydrase assay method. Highly purified CA isoforms were employed, for which the kinetic parameters for the physiologic reaction (CO_2_ hydration) were measured (see the Experimental section for details), monitoring the color change produced by the formation of H^+^ ions (and bicarbonate). For all the pure enzymes, the kinetic parameters (k_cat_ and k_cat_/K_M_) are measured and these values are given in the Table 2. These activities were highly inhibited by the clinically used sulfonamide inhibitor acetazolamide (AAZ), as shown in Table 2. It was observe that all these enzymes are highly efficient catalysts with k_cat_/K_M_ > 10^7^ M^−1^x s^−1^.

The following structure-activity relationship can be inferred from the inhibition data of compounds **6a–o** (Table 3).

I Tthe cytosolic hCA II isoform was strongly inhibited by all the synthesized compounds **6a–o**, in a low to medium nanomolar range (K_i_s = 7.7 nM to 0.2527 µM). The best activity against hCA II was shown by compound **6i** (K_i_ = 7.7 nM), possessing a fluoro group attached at the 5th position of the indole ring and an isopropyl group anchored to the nitrogen of indole. It was almost twofold more active than the standard AAZ (K_i_ = 12.1 nM). Compounds **6d–6g**, were found to have potent activity at the nanomolar concentration against hCA II, with K_i_ ranging from 20.9 to 63.9 nM. Compounds **6k–6o**, containing a chloro group at the 5th position of indole, showed lower activity in the range of 61.7 to 252.7 nM, compared to compounds containing a fluoro group and unsubstituted indole.
II The transmembrane hCA IX isoform, which is expressed exclusively in tumors, was also strongly inhibited by the synthesized compounds in the medium nanomolar range (K_i_s = 34.9 nM to 0.3246 µM). Compounds **6d**, **6e**, **6f**, and **6i** showed equipotent nanomolar activity with AAZ, with K_i_s ranging from 34.9 nM to 41.3 nM. Among these compounds, **6i** showed the best activity (K_i_ = 34.9 nM) against hCA IX isoform.
III The cytosolic hCA I and hCA XIII isoforms were inhibited by all synthesized compounds in the high nanomolar range (K_i_s > 500 nM). However, compounds **6b** and **6d** showed moderate activity with K_i_s of 69.8 nM and 65.8 nM respectively against hCA XIII isoform.

From the above structure–activity relationship, it was found that compound **6i** was the most potent compound with a K_i_ values of 7.7 nM against hCAII and 34.9 nM against hCA IX.

## 3. Conclusions

In conclusion, we report here the synthesis of a series of 1,2,3-triazole-linked triazino[5,6-b]indole-benzene sulfonamide hybrids (**6a–6o**). The structures of these compounds where confirmed by different spectral and elemental analyses methods (Supplemental Data S1). The Biological evaluation of sulfonamides was performed against hCA I, hCA II, hCA IX, and hCA XIII. All compounds showed low to moderate inhibitory activity against hCA II and hCA IX isoforms, at concentrations in the range between 7.7 nM and 0.3246 µM. Compound **6i** emerged as a potent hCA II and hCA IX inhibitor (K_i_ = 7.7 nM against hCA II and 34.9 nM against hCA IX). The compounds **6b** and **6d** was showed activity at medium nanomolar concentrations, with K_i_ of 69.8 nM and 65.8 nM, respectively, against hCA XIII isoform. Thus, the compound **6i** can be emerged as a novel potential lead compound to develop selective carbonic anhydrase inhibitors against the hCA II isoform.

## 4. Experimental Section

### 4.1. General Experimental Conditions

All the chemicals and solvents utilized as obtained from the suppliers. Wherever necessary, anhydrous solvents are used. Thin-layer chromatography analysis (TLC), was carried-out by utilizing Merck silica gel 60 F_254_ aluminum plates. A Stuart Digital Melting point Apparatus (SMP 30) was used to determining the melting point of the compounds, which were uncorrected. The ^1^H and ^13^C NMR spectra were recorded on Bruker Avance 500 MHz and 125 MHz respectively, with DMSO-*d*_6_ as the solvent. The chemical shift values were calculated in ppm using TMS as the standard reference. The HRMS were recorded on Agilent QTOF mass spectrometer 6540 series and were performed using ESI techniques at 70 eV. All the newly synthesized analogs were evaluated in vitro for their inhibitory activity against a panel of recombinant CA isoforms, i.e., hCA I, hCA II, hCA IX, and hCA XIII, obtained inhouse, using the stopped-flow CO_2_ hydrase assay.

#### 4.1.1. Synthesis of *N*-Alkylated isatins (**3a–o**)

To a stirred solution of isatin (0.5 g, 0.00398 mole) in DMF (10 mL) we added potassium carbonate (0.939 g, 0.006796 mole) and potassium iodide (0.05 mole%), and the resulting solution was stirred for about 30 min. After the specified time interval, the respective alkyl halides (**2a–e**) was added and the resulting solution was allowed to reflux. The progress of the reaction was monitored by using TLC. Upon the completion of the reaction as assessed by TLC, the reaction mixture was poured into ice water, and the precipitated solid was collected, washed with water, and recrystallized from ethanol to yield compounds (**3a–o**) (yield 72–75%) [31,32].

#### 4.1.2. Synthesis of *N*-Alkylated triazino[5,6-b]indolethioether derivatives (**4a–o**) 

To a stirred solution of *N*-alkylated isatin (0.450 g, 0.002378 mol) in 40% aqueous 1,4-dioxane (5 mL), thiosemicarbazide (0.260 g, 0.00285 mol) and Cs_2_CO_3_ (0.720 g, 0.00285 mol) were added. The resulting solution was refluxed overnight. Upon completion of the reaction (as determined by TLC), the reaction mixture was cooled to rt. The solid byproducts were filtered off and the filtrate was acidified with conc. HCl to pH 1–3. The obtained solids were collected washed with water and dried to give yellow-colored solids which were used without any further purification (yield 68–70%) [31,32].

#### 4.1.3. Synthesis of *N*-Alkyl-3-prop-2-yn-1-ylthio)-5*H*-[1,2,4]triazino[5,6b]indole derivatives (**5a–o**)

To a stirred solution of compounds **4a–o** (0.140 g, 0.00607 mole) in DMF (3 mL), K_2_CO_3_ (0.101 g, 0.000729 mol) and propargyl bromide (0.087 g, 0.000729 mol) were added. The resulting reaction mixture was stirred at rt overnight. Upon completion of the reaction (monitored by TLC), the reaction mixture was poured in ice-cold water, and the formed solid was collected, washed with water, and dried to give brown-colored solids that were used without any further purification (yield 86–90%).

#### 4.1.4. Synthesis of 4-(4-(*N*-Alkyl-5*H*-[1,2,4]triazino[5,6b]indol-3-yl)thio)methyl)- 1*H*-1,2,3-triazol-1-yl)benzenesulfonamide derivatives (**6a–o**)

The compounds **5a–o** (0.04 g, 0.0001 mol) and 4-azido benzene sulfonamide (0.024 g, 0.000 mol) were suspended in 2 mL of a 1:1 water/tert-butanol mixture. Sodium ascorbate (0.048 g, 0.0002 mol) was added, followed by copper (II) sulfate pentahydrate (0.031 g, 0.0001 mol). The heterogeneous mixture was stirred vigorously overnight at which point it cleared and TLC analysis indicated complete consumption of the starting materials. The reaction mixture were diluted with water and cooled in ice to obtained the brown precipitate, which were collected by filtration. After washing with cold water the precipitate were dried under vacuum to afford a pure product as a brown amorphous solid (**6a–o**).

*4-(4-(((5-methyl-5H-[1,2,4]triazino[5,6b]indol-3-yl)thio)methyl-1H-1,2,3-triazol-1-yl)benzene sulfonamide* (**6a**) Yield: 57%; Color: Brown solid; mp: 235–240 °C; ^1^H NMR (500 MHz, DMSO-*d_6_*) *δ* 8.87 (s, 1H), 8.34 (s, 1H), 8.10 (s, 2H), 7.99 (s, 2H), 7.80 (s, 2H), 7.51 (s, 3H), 4.77 (s, 2H), 3.86 (s, 3H);13C NMR (125 MHz, DMSO) δ 167.1, 159.9, 158.0, 147.2, 145.7, 144.2, 141.3, 139.0, 138.5, 127.9, 122.5, 120.8, 113.2, 108.1, 107.9, 28.1, 25.29. HR-MS (ESI-QTOF): *m/z* calculated for [M + H]^+^ C_19_H_16_N_8_O_2_S_2_; 453.0916; found 453.0965.

*4-(4-(((5-ethyl-5H-[1,2,4]triazino[5,6b]indol-3-yl)thio)methyl-1H-1,2,3-triazol-1-yl)benzene sulfonamide* (**6b**) Yield: 59%; Color: Brown solid; mp: 202–204 °C; ^1^H NMR (500 MHz, DMSO-*d_6_*) *δ*8.85 (s, 10H), 8.38 (dd, *J* = 17.8, 4.8 Hz, 11H), 8.10 (d, *J* = 8.6 Hz, 21H), 7.99 (d, *J* = 8.6 Hz, 20H), 7.85 (d, *J* = 8.2 Hz, 8H), 7.83–7.75 (m, 12H), 7.50 (s, 20H), 4.75 (s, 19H), 4.44 (q, *J* = 7.0 Hz, 21H), 1.35 (t, *J* = 7.2 Hz, 28H); ^13^C NMR (125 MHz, DMSO-*d_6_*) *δ* 166.58, 146.11, 145.83, 144.23, 141.55, 141.02, 139.02, 131.43, 127.91, 123.28, 122.37, 122.06, 120.72, 117.94, 111.68, 36.45, 25.31, 13.73; HR-MS (ESI-QTOF): *m/z* calculated for [M + H]^+^ C_20_H_18_N_8_O_2_S_2_; 467.1072; found 467.1120.

*4-(4-(((5-propyl-5H-[1,2,4]triazino[5,6b]indol-3-yl)thio)methyl-1H-1,2,3-triazol-1-yl)benzene sulfonamide* (**6c**) Yield: 61%; Color: Brown solid; mp: 261–263 °C; ^1^H NMR (500 MHz, DMSO) δ 8.62 (s, 1H), 8.13 (s, 1H), 7.81 (d, *J* = 56.5 Hz, 5H), 7.57 (d, *J* = 35.0 Hz, 3H), 7.27 (s, 3H), 4.51 (s, 2H), 4.12 (s, 2H), 1.57 (s, 3H); ^13^C NMR (125 MHz, DMSO) δ 166.7, 146.6, 144.2, 141.4, 139.0, 131.4, 127.9, 123.2, 122.3, 121.9, 120.7, 117.9, 111.8, 43.0, 25.35, 21.62, 11.6.HR-MS (ESI-QTOF): *m/z* calculated for [M + H]^+^ C_21_H_20_N_8_O_2_S_2_; 481.1229; found 481.1268.

*4-(4-(((5-isopropyl-5H-[1,2,4]triazino[5,6b]indol-3-yl)thio)methyl-1H-1,2,3-triazol-1-yl)benzene sulfonamide* (**6d**) Yield: 53%; Color: Brown solid; mp: 213–215 °C; ^1^H NMR (500 MHz, DMSO-*d_6_*) *δ* 8.84 (s, 1H), 8.19 (d, *J* = 7.5 Hz, 1H), 8.09 (d, *J* = 8.4 Hz, 2H), 8.04–7.93 (m, 4H), 7.62 (t, *J* = 8.1 Hz, 1H), 7.49 (s, 2H), 5.21–5.13 (m, 1H), 4.73 (s, 2H), 1.61 (d, *J* = 6.7 Hz, 6H). ^13^C NMR (125 MHz, DMSO) δ 166.2, 146.2, 145.9, 144.2, 141.5, 140.6, 139.0, 131.3, 127.9, 123.1, 122.3, 122.0, 120.7, 118.2, 112.6, 46.7, 25.3, 20.3. HR-MS (ESI-QTOF): *m/z* calculated for [M + H]^+^ C_21_H_20_N_8_O_2_S_2_; 481.1229; found 481.1294.

*4-(4-(((5-allyl-5H-[1,2,4]triazino[5,6b]indol-3-yl)thio)methyl-1H-1,2,3-triazol-1-yl)benzene sulfonamide* (**6e**) Yield: 61%; Color: Brown solid; mp: 230–232 °C; ^1^H NMR (500 MHz, DMSO-*d_6_*) *δ*8.84 (s, 1H), 8.39 (s, 1H), 8.05 (d, *J* = 45.9 Hz, 5H), 7.78 (s, 2H), 7.52 (s, 2H), 6.02 (s, 1H), 5.08 (s, 4H), 4.77 (s, 2H); ^13^C NMR (125 MHz, DMSO) δ 166.4, 147.0, 146.5, 144.3, 141.5, 141.3, 139.0, 131.4, 123.5, 122.5, 122.0, 120.8, 117.9, 112.0, 43.5, 25.3. HR-MS (ESI-QTOF): *m/z* calculated for [M + H]^+^ C_21_H_10_N_8_O_2_S_2_; 479.1072; found 479.1087.

*4-(4-(((8-fluoro-5-methyl-5H-[1,2,4]triazino[5,6b]indol-3-yl)thio)methyl-1H-1,2,3-triazol-1-yl)benzene sulfonamide* (**6f**) Yield: 59%; Color: Brown solid; mp: 244–246 °C; ^1^H NMR (500 MHz, DMSO-*d_6_*) *δ* 8.86 (s, 1H), 8.20–8.16 (m, 1H), 8.10 (d, *J* = 8.6 Hz, 2H), 7.99 (d, *J* = 8.6 Hz, 2H), 7.84 (dd, *J* = 8.8, 3.9 Hz, 1H), 7.70–7.63 (m, 3H), 7.51 (s, 1H), 4.76 (s, 2H), 3.86 (s, 3H); ^13^C NMR (125 MHz, DMSO-*d_6_*) *δ* 167.15, 159.89, 147.16, 144.25, 139.02, 138.48, 127.90, 122.49, 120.78, 118.93, 118.73, 118.67, 118.59, 113.14, 107.91, 28.04, 25.29; HR-MS (ESI-QTOF): *m/z* calculated for [M + H]^+^ C_19_H_15_FN_8_O_2_S_2_;471.0822; found 471.0824.

*4-(4-(((8-fluoro-5-ethyl-5H-[1,2,4]triazino[5,6b]indol-3-yl)thio)methyl-1H-1,2,3-triazol-1-yl)benzene sulfonamide* (**6g**) Yield: 53%; Color: Brown solid; mp: 246–248 °C; ^1^H NMR (500 MHz, DMSO-*d_6_*) *δ* 8.85 (s, 1H), 8.19 (dd, *J* = 8.1, 2.3 Hz, 1H), 8.10 (d, *J* = 8.7 Hz, 2H), 7.99 (d, *J* = 8.7 Hz, 2H), 7.91 (dd, *J* = 8.9, 4.0 Hz, 1H), 7.66 (td, *J* = 9.2, 2.4 Hz, 1H), 7.50 (s, 2H), 4.75 (s, 2H), 4.45 (q, *J* = 7.0 Hz, 2H), 1.34 (t, *J* = 7.1 Hz, 3H); ^13^C NMR (125 MHz, DMSO-*d_6_*) *δ* 167.16, 159.85, 146.63, 145.75, 144.26, 139.03, 137.40, 127.91, 122.39, 120.73, 118.97, 118.87, 118.77, 113.24, 108.09, 36.63, 25.33, 13.73; HR-MS (ESI-QTOF): *m/z* calculated for [M + H]^+^ C_20_H_17_FN_8_O_2_S_2_; 485.0978; found 485.0979.

*4-(4-(((8-fluoro-5-propyl-5H-[1,2,4]triazino[5,6b]indol-3-yl)thio)methyl-1H-1,2,3-triazol-1-yl)benzene sulfonamide* (**6h**) Yield: 63%; Color: Brown solid; mp: 240–242 °C; ^1^H NMR (500 MHz, DMSO) δ 8.84 (s, 1H), 8.18 (dd, *J* = 8.2, 2.5 Hz, 1H), 8.09 (d, *J* = 8.8 Hz, 2H), 7.98 (d, *J* = 8.8 Hz, 2H), 7.89 (dd, *J* = 9.0, 4.0 Hz, 1H), 7.64 (td, *J* = 9.2, 2.6 Hz, 1H), 7.50 (s, 2H), 4.74 (s, 2H), 4.35 (t, *J* = 6.9 Hz, 2H), 1.83–1.74 (m, 2H), 0.78 (t, *J* = 7.4 Hz, 3H); ^13^C NMR (125 MHz, DMSO) δ 167.3, 159.8, 157.9, 147.1, 145.8, 144.3, 141.1, 139.0, 137.8, 127.9, 122.3, 120.7, 113.4, 108.2, 107.9, 43.2, 25.4, 21.6, 11.5. HR-MS (ESI-QTOF): *m/z* calculated for [M + H]^+^ C_21_H_19_ FN_8_O_2_S_2_ 499.1135; found 499.1153.

*4-(4-(((8-fluoro-5-isopropyl-5H-[1,2,4]triazino[5,6b]indol-3-yl)thio)methyl-1H-1,2,3-triazol-1-yl)benzene sulfonamide* (**6i**) Yield: 55%; Color: Brown solid; mp: 235–237 °C; ^1^H NMR (500 MHz, DMSO-*d_6_*) *δ* 8.84 (s, 1H), 8.20 (d, *J* = 7.8 Hz, 1H), 8.10 (d, *J* = 8.0 Hz, 2H), 7.98 (d, *J* = 8.3 Hz, 3H), 7.63 (s, 1H), 7.50 (s, 2H), 5.17 (s, 1H), 4.73 (s, 2H), 1.61 (d, *J* = 6.1 Hz, 6H); ^13^C NMR (125 MHz, DMSO) δ 166.8, 159.7, 157.8, 146.7, 145.8, 144.2, 141.1, 139.0, 137.0, 127.9, 122.3, 120.7, 119.2, 118.8, 46.9, 25.3, 20.3. HR-MS (ESI-QTOF): *m/z* calculated for [M + H]^+^ C_21_H_19_ FN_8_O_2_S_2_499.1135; found 499.1174.

*4-(4-(((8-fluoro-5-allyl-5H-[1,2,4]triazino[5,6b]indol-3-yl)thio)methyl-1H-1,2,3-triazol-1-yl)benzene sulfonamide* (**6j**) Yield: 51%; Color: Brown solid; mp: 245–247 °C; ^1^H NMR (500 MHz, DMSO-*d_6_*) *δ* 8.81 (s, 1H), 8.21 (d, *J* = 6.7 Hz, 1H), 8.09 (d, *J* = 8.3 Hz, 2H), 7.99 (d, *J* = 8.3 Hz, 2H), 7.78 (d, *J* = 5.0 Hz, 1H), 7.65 (t, *J* = 8.2 Hz, 1H), 7.50 (s, 2H), 6.06-5.96 (m, 1H), 5.13–5.05 (m, 4H), 4.74 (s, 2H); ^13^C NMR (125 MHz, DMSO) δ 167.4, 159.9, 158.0, 146.9, 145.7, 144.3, 141.3, 139.0, 137.6, 132.1, 127.9, 122.4, 120.8, 119.0, 118.7, 113.6, 108.3, 43.8, 25.3. HR-MS (ESI-QTOF): *m/z* calculated for [M + H]^+^ C_21_H_17_FN_8_O_2_S_2_ 497.0978; found 497.1027.

*4-(4-(((8-chloro-5-methyl-5H-[1,2,4]triazino[5,6b]indol-3-yl)thio)methyl-1H-1,2,3-triazol-1-yl)benzene sulfonamide* (**6k**) Yield: 62%; Color: Brown solid; mp: 243–248 °C; ^1^H NMR (500 MHz, DMSO) δ 8.87 (s, 1H), 8.39 (s, 1H), 8.10 (d, *J* = 8.1 Hz, 1H), 7.99 (d, *J* = 8.1 Hz, 1H), 7.84 (d, *J* = 5.5 Hz, 1H), 7.51 (s, 1H), 4.77 (s, 1H), 3.86 (s, 1H);^13^C NMR (126 MHz, CDCl_3_) δ 172.04, 151.12, 150.44, 148.97, 145.57, 144.27, 143.71, 135.73, 132.74, 127.25, 126.31, 125.47, 124.14, 118.20, 109.43, 41.18, 29.99. HR-MS (ESI-QTOF): *m/z* calculated for [M + H]^+^ C_19_H_15_ClN_8_O_2_S_2_ 487.0526; found 487.0530.

*4-(4-(((8-chloro-5-ethyl-5H-[1,2,4]triazino[5,6b]indol-3-yl)thio)methyl-1H-1,2,3-triazol-1-yl)benzene sulfonamide* (**6l**) Yield: 57%; color: Brown solid; mp: 252–256 °C; ^1^H NMR (500 MHz, DMSO) δ 8.86 (s, 1H), 8.40 (s, 1H), 8.10 (d, *J* = 7.3 Hz, 1H), 7.99 (d, *J* = 7.0 Hz, 1H), 7.91 (d, *J* = 7.6 Hz, 1H), 7.82 (s, 1H), 7.51 (s, 1H), 4.75 (s, 1H), 4.44 (d, *J* = 5.7 Hz, 1H), 1.29 (d, *J* = 47.6 Hz, 1H); ^13^C NMR (126 MHz, CDCl_3_) δ 172.04, 151.80, 150.30, 149.06, 145.56, 145.35, 143.81, 132.66, 132.51, 127.17, 126.07, 125.52, 123.87, 122.79, 118.21, 53.69, 32.67, 29.99.HR-MS (ESI-QTOF): *m/z* calculated for [M + H]^+^ C_20_H_18_ClN_8_O_2_S_2_ 501.0682; found 501.0694.

*4-(4-(((8-chloro-5-propyl-5H-[1,2,4]triazino[5,6b]indol-3-yl)thio)methyl-1H-1,2,3-triazol-1-yl)benzene sulfonamide* (**6m**) Yield: 63%; Color: Brown solid; mp: 257–262 °C; ^1^H NMR (500 MHz, DMSO) δ 8.85 (s, 1H), 8.39 (s, 1H), 8.10 (d, *J* = 7.1 Hz, 1H), 7.99 (d, *J* = 7.0 Hz, 1H), 7.90 (d, *J* = 7.9 Hz, 1H), 7.80 (d, *J* = 7.3 Hz, 1H), 7.51 (s, 1H), 4.74 (s, 1H), 4.35 (s, 1H), 1.78 (d, *J* = 6.1 Hz, 1H), 0.79 (s, 2H); ^13^C NMR (126 MHz, DMSO) δ 167.46, 146.95, 145.67, 144.25, 140.63, 139.99, 138.86, 131.10, 127.90, 127.75, 122.34, 121.44, 120.70, 119.24, 113.68, 43.18, 25.42, 21.48, 11.56.HR-MS (ESI-QTOF): *m/z* calculated for [M + H]^+^ C_21_H_20_ClN_8_O_2_S_2_ 515.0839; found 515.0839.

*4-(4-(((8-chloro-5-isopropyl-5H-[1,2,4]triazino[5,6b]indol-3-yl)thio)methyl-1H-1,2,3-triazol-1-yl)benzene sulfonamide* (**6n**) Yield: 56%; Color: Brown solid; mp: 263–265 °C; ^1^H NMR (500 MHz, DMSO) δ 8.85 (s, 1H), 8.40 (s, 1H), 8.10 (d, *J* = 8.6 Hz, 1H), 7.99 (d, *J* = 8.6 Hz, 1H), 7.95 (d, *J* = 8.9 Hz, 1H), 7.80–7.75 (m, 1H), 7.50 (s, 1H), 5.16 (dt, *J* = 13.6, 6.7 Hz, 1H), 4.73 (s, 1H), 1.61 (d, *J* = 6.8 Hz, 1H);^13^C NMR (126 MHz, DMSO) δ 166.92, 146.52, 145.82, 144.26, 140.54, 139.11, 139.01, 130.89, 127.92, 127.53, 122.36, 121.33, 120.73, 119.71, 114.31, 47.03, 30.95, 25.38, 20.33.HR-MS (ESI-QTOF): *m/z* calculated for [M + H]^+^ C_21_H_20_ClN_8_O_2_S_2_ 515.0839; found 515.0856.

*4-(4-(((8-chloro-5-allyl-5H-[1,2,4]triazino[5,6b]indol-3-yl)thio)methyl-1H-1,2,3-triazol-1-yl)benzene sulfonamide* (**6o**) Yield: 67%; Color: Brown solid; mp: 261–264 °C; ^1^H NMR (500 MHz, TFE) δ 8.81 (s, 1H), 8.40 (s, 1H), 8.08 (d, *J* = 8.7 Hz, 1H), 7.98 (d, *J* = 8.7 Hz, 1H), 7.81–7.75 (m, 1H), 7.50 (s, 1H), 5.99 (ddd, *J* = 22.0, 10.3, 5.0 Hz, 1H), 5.13–5.02 (m, 2H), 4.73 (s, 1H);^13^C NMR (126 MHz, DMSO) δ 167.54, 146.67, 145.70, 144.27, 140.68, 139.65, 138.99, 131.82, 131.04, 127.89, 122.51, 121.44, 120.76, 119.42, 117.79, 113.91, 43.48, 31.33, 25.06.HR-MS (ESI-QTOF): *m/z* calculated for [M + H]^+^ C_21_H_17_ClN_8_O_2_S_2_ 513.0682; found 513.069.

### 4.2. CA Inhibition

An SX.18 V-R Applied Photophysics (Oxford, UK) stopped-flow instrument was used to assay the catalytic inhibition of various CA enzymes [32]. Phenol Red (at a concentration of 0.2 mM) was used as an indicator, working at maximum absorbance of 557 nm, with 10 mM Hepes (pH 7.4) buffer, 0.1 M Na_2_SO_4_ (for maintaining constant ionic strength; these anions are not inhibitory in the used concentration), following the CA-catalyzed CO_2_ hydration reaction for a period of 5–10 s. Saturated CO_2_ solutions in water at 25 °C were used as a substrate. Stock solutions of the inhibitors were prepared at a concentration of 10 mM (in DMSO/Water 1:1, *v/v*), and dilutions up to 0.01 nM were prepared using the assay buffer mentioned above. At least 7 different inhibitor concentrations were used for measuring the inhibition constant. Inhibitor (I) and enzyme (E) solutions were pre-incubated together for 10 min at room temperature prior to the assay, in order to allow for the formation of the E–I complex. IC_50_-s values were calculated from the enzyme activity (as k_cat_, see Table 2) with respect to the inhibitor concentration. Triplicate experiments were done for each inhibitor concentration, and the values reported in this paper are the mean of such results. The inhibition constants were obtained by non-linear least-square methods, using the Cheng–Prusoff equation, as reported earlier, and represent the mean from at least three different determinations: K_I_ = IC_50_/[(1 + [S]/K_M_)]. K_M_ values for all enzymes were reported earlier (see Table 2) by us [26,27,28,29,30,31]; [S] is the CO_2_ concentration at which the experiments were performed. All CA isozymes used here were recombinant proteins obtained as reported earlier by our group [33,34,35,36,37,38].

## Figures and Tables

**Table 1:** Carbonic anhydrase (CA) isoforms and associated diseases.

Disease	Isoform Target
Glaucoma	CA II, CA IV, CA XII
Cancer	CA IX, CA XII
Epilepsy	CA VII
Antineuropathic pain	CA VII
Obesity	CA VA

**Table 2:** Kinetic parameters of the pure CA isoforms employed in this work and inhibition constants for acetazolamide (AAZ), a standard sulfonamide drug.

Organisms	CA Class	Acronym	K_cat_ (s^−1^)	k_cat_/K_M_ (M^−1^ × s^−1^)	K_I_ (Acetazolamide) (nM)
*Homo sapiens*	α	hCA I	2.0 × 10^5^	5.0 × 10^7^	250
	α	hCA II	1.4 × 10^6^	1.5 × 10^8^	12.1
	α	hCA IX ^a^	3.8 × 10^5^	5.5 × 10^7^	25.8
	α	hCA_XIII	1.5 × 10^5^	1.1 × 10^7^	17.0

^a^ Catalytic domain.

**Table 3:** Inhibition of hCA isoforms I, II, IX, and XIII with compounds **6a–o** and AAZ as a standard inhibitor.

		K_I_ (nM)			
Compound	Structure	hCA I	hCA II	hCA IX	hCA XIII
**6a**	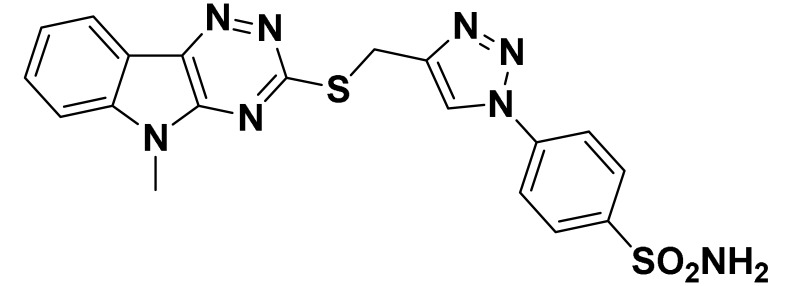	910.1	65.5	285.6	77.8
**6b**	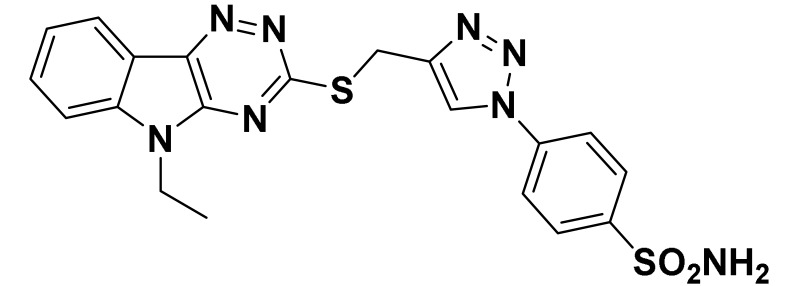	642.2	72.0	172.5	69.8
**6c**	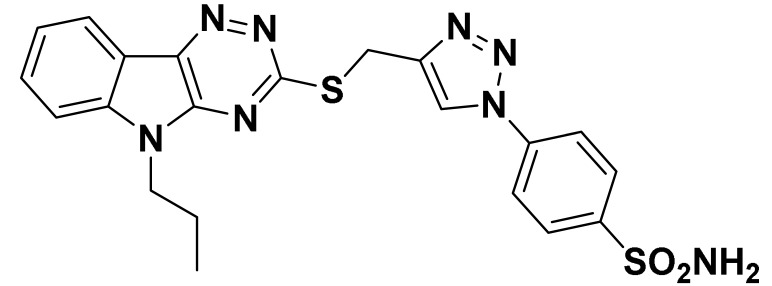	3960	88.7	219.4	364.8
**6d**	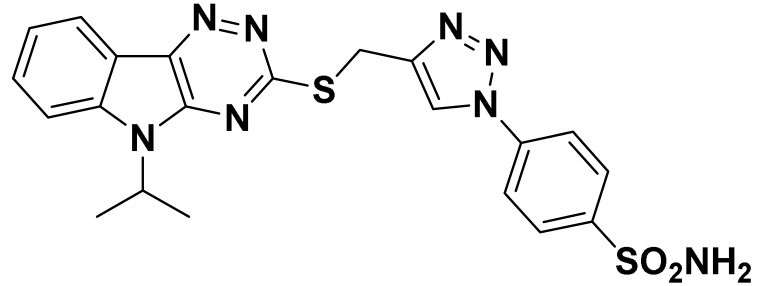	314.7	20.9	37.8	65.8
**6e**	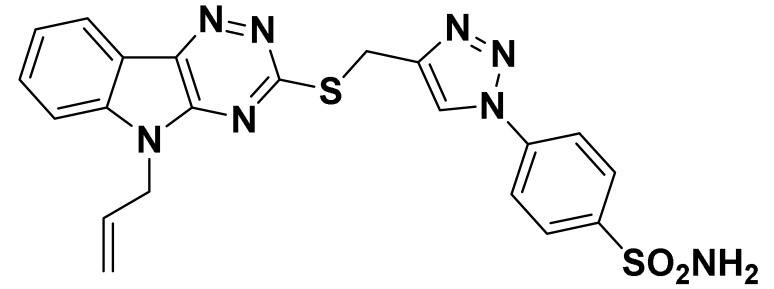	535.8	41.7	41.1	626.7
**6f**	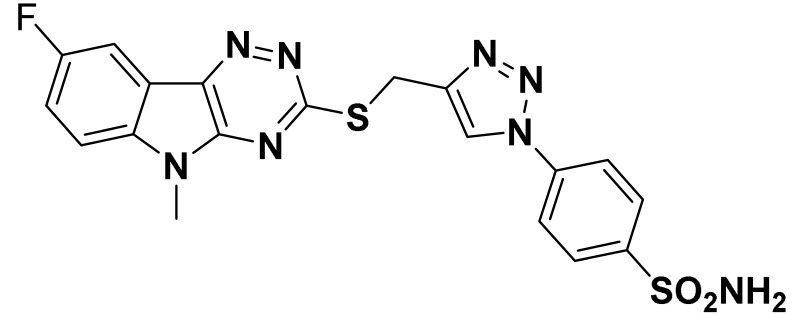	766.2	59.6	41.3	834.8
**6g**	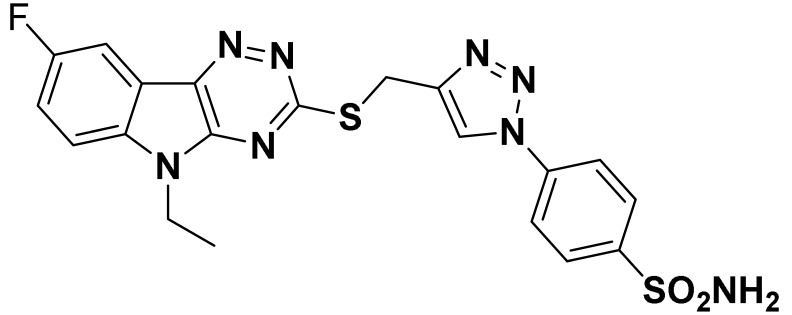	698.5	63.9	193.1	675.0
**6h**	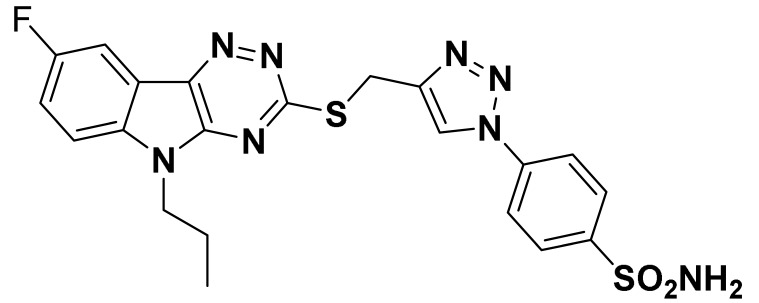	764.2	682.7	118.6	1815
**6i**	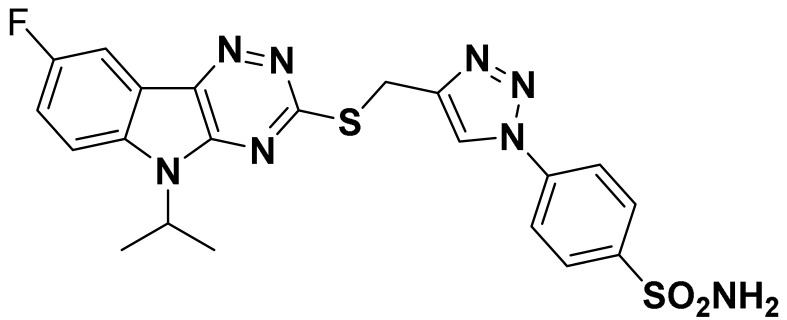	379.2	7.7	34.9	736.2
**6j**	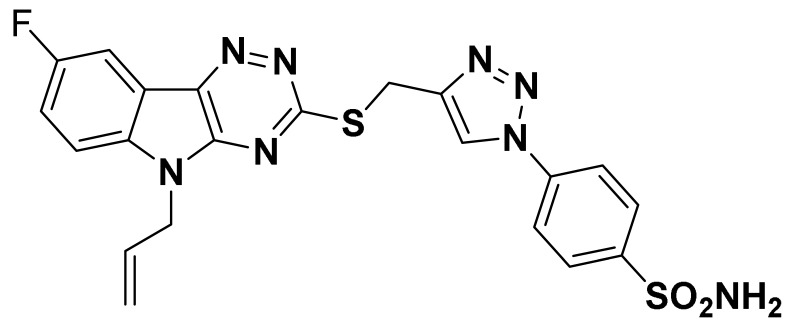	4592	73.7	401.7	793.6
**6k**	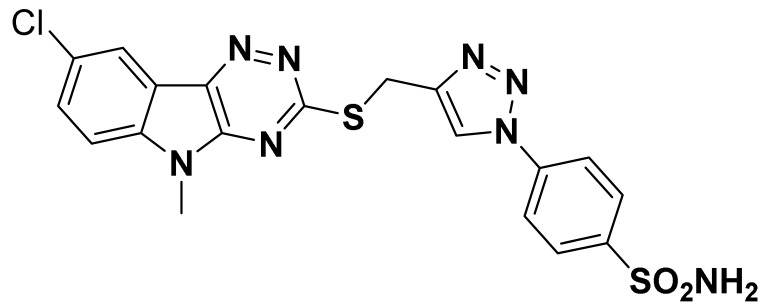	5140	252.7	330.7	867.0
**6l**	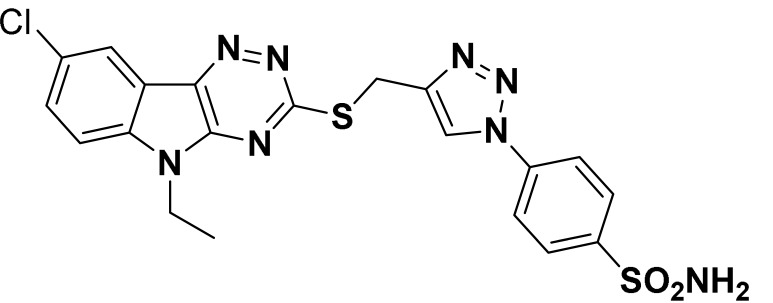	2837	184.6	150.4	3980
**6m**	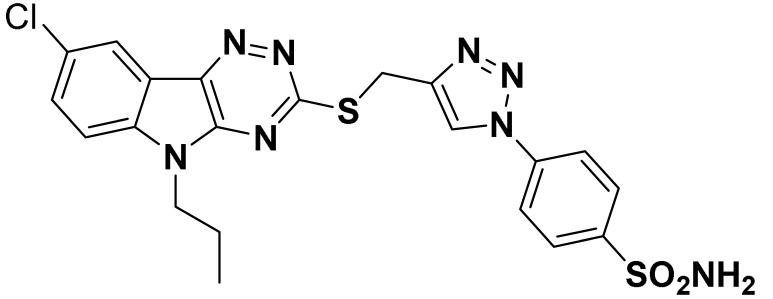	6513	61.7	204.5	823.8
**6n**	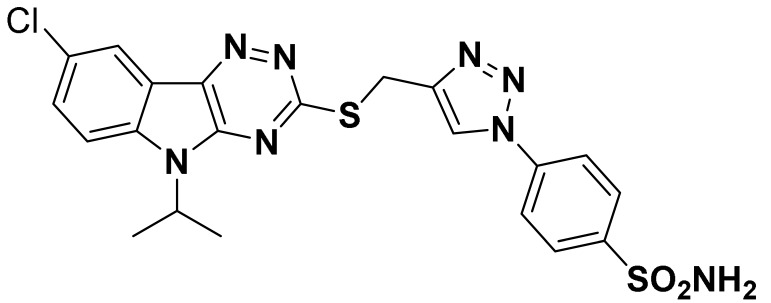	571.9	179.2	320.8	91.3
**6o**	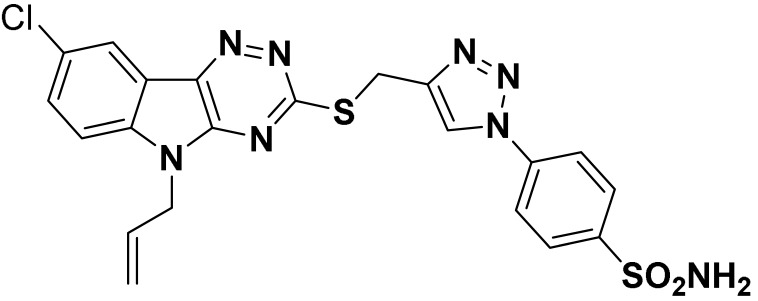	694.4	97.1	324.6	2300
**AAZ**		250.0	12.1	25.8	17.0

## Supplementary Materials

The spectral data as supporting information are available online at https://www.mdpi.com/2218-1989/10/5/200/s1; Data S1: 1H NMR spectra of 6a–o, 13C NMR spectra of 6a–o.

## References

[1] Alterio V., Di Fiore A., Ambrosio K.D., Supuran C.T., de Simone G. (2012). Multiple binding modes of inhibitors to carbonic anhydrases: How to design specific drugs targeting 15 different isoforms?. Chem. Rev..

[2] Supuran C.T. (2008). Carbonic anhydrases: Novel therapeutic applications for inhibitors and activators. Nat. Rev. Drug Discov..

[3] Supuran C.T. (2016). Structure and function of carbonic anhydrases. Biochem. J..

[4] Supuran C.T. (2018). Carbonic anhydrases and metabolism. Metabolites.

[5] Jensen E.L., Clement R., Kosta A., Maberly S.C., Gontero B. (2019). A new wide spread sub class of carbonic anhydrase in marine phytoplankton. ISME J..

[6] Lock F.E., McDonald P.C., Lou Y., Serrano I., Chafe S.C., Ostlund C., Aparicio S., Winum J.Y., Supuran C.T., Dedhar S. (2013). Targeting carbonic anhydrase IX depletes breast cancer stem cells within the hypoxic niche. Oncogene.

[7] Bayram E., Senturk M., Kufrevioglu O.I., Supuran C.T. (2008). In vitro inhibition of salicylic acid derivatives on human cytosolic carbonic anhydrase isozymes I and II. Bioorg. Med. Chem..

[8] Bozdag M., Pinard M., Carta F., Masini E., Scozzafava A., McKenna R., Supuran C.T. (2014). A Class of 4-Sulfamoylphenyl-ω-aminoalkyl Ethers with Effective Carbonic Anhydrase Inhibitory Action and Antiglaucoma Effects. J. Med. Chem..

[9] Chegaev K., Lazzarato L., Tamboli Y., Boschi D., Blangetti M., Scozzafava A., Carta F., Masini E., Fruttero R., Supuran C.T. (2014). Furazan and furoxan sulfonamides are strong α-carbonic anhydrase inhibitors and potential anti-glaucoma agents. Bioorg. Med. Chem..

[10] De Luca L., Ferro S., Damiano F.M., Supuran C.T., Vullo D., Chimirri A., Gitto R. (2014). Structure-based screening for the discovery of new carbonic anhydrase XII inhibitors, *Eur*. J. Med. Chem..

[11] Bruno E., Buemi M.R., De Luca L., Ferro S., Monforte A.M., Supuran C.T., Vullo D., De Sarro G., Russo E., Gitto R. (2016). In Vivo Evaluation of Selective Carbonic Anhydrase Inhibitors as Potential Anticonvulsant Agents. ChemMedChem.

[12] Scozzafava A., Menabuoni L., Mincione F., Supuran C.T. (2002). Carbonic anhydrase inhibitors: A general approach for the preparation of water-soluble sulfonamides incorporating polyamino-polycarboxylate tails and of their metal complexes possessing long-lasting, Topical intraocular pressure-lowering properties. J. Med. Chem..

[13] Tars K., Vullo D., Kazaks A., Leitans J., Lends A., Grandane A., Zalubovskis A., Scozzafava A., Supuran. C.T. (2013). Sulfocoumarins (1,2-Benzoxathiine-2,2-dioxides): A Class of Potent and Isoform-Selective Inhibitors of Tumor-Associated Carbonic Anhydrases. J. Med. Chem..

[14] Maresca A., Scozzafava A., Supuran C.T. (2010). 7,8-Disubstituted-but not 6,7-disubstituted coumarins selectively inhibit the transmembrane, tumor associated carbonic anhydrase IX and XII over the cytosolic ones I and II in the low nanomolar/subnanomolar range. Bioorg. Med. Chem. Lett..

[15] Innocenti A., Sarıkaya S.B.O., Gulcin I., Supuran C.T. (2010). Carbonic anhydrase inhibitors, Inhibition of mammalian isoforms I-XIV with a series of natural product polyphenols and phenolic acids. Bioorg. Med. Chem..

[16] Pinard M.A., Mahon B., McKenna R. (2015). Probing the surface of human carbonicanhydrase for clues towards the design of isoform specific inhibitors. Biomed. Res. Int..

[17] Aggarwal M., Kondeti B., McKenna R. (2013). Insights towards sulfonamide drug specificity in alpha-carbonic anhydrases. Bioorg. Med. Chem..

[18] Kgokong J.L., Smith P.P., Matsabisa G.M. (2005). 1,2,4-Triazino-[5,6b]indole derivatives: Effects of trifluoromethyl group on in-vitro antimalarial activity. Bioorg. Med. Chem..

[19] Rahim F., Ullah K., Ullah H. (2015). Triazinoindole analogs as potent inhibitors of α-glucosidase: Synthesis, biological evaluation and molecular docking studies. Bioorg. Med. Chem..

[20] Shelke S.M., Bhosale S.H. (2010). Synthesis, antidepressant evaluation and QSAR studies of novel 2-(5H-[1,2,4]triazino[5,6b]indol3-ylthio)-N-(substituted phenyl)acetamides. Bioorg. Med. Chem. Lett..

[21] Monge A., Palop J.A., Ramierz C., Font M., Fernandez A.E. (1991). New 5H-1,2,4-triazino[5,6b]indole and aminoindole derivatives. Synthesis and studies as inhibitors of blood platelet aggregation, anti-hypertensive agents and thromboxane synthetase inhibitors. Eur. J. Med. Chem..

[22] Aswar U.M., Kalshetti P.P., Shelke S.M., Bhosale S.H., Bodhankar S.L. (2012). Effect of newly synthesized 1,2,4-triazino[5,6b]indole-3-thione derivatives on olfactory bulbectomy induced depression in rats. Asian. J. Trop. Biomed..

[23] Tomchin A.B., Uryupov O.Y., Zhukova T.I., Kuznetsova T.A. (1997). Thiourea and thiosemicarbazide derivatives: Structure, transformations, and pharmacological activity. Part III. Antihypoxic and anti-inflammatory activity of 1,2,4-triazino[5,6b]indole derivatives. Pharma. Chem. J..

[24] Demir-Yazici K., Bua S., Supuran C.T. (2019). Indole-Based Hydrazones Containing A Sulfonamide Moiety as Selective Inhibitors of Tumor-Associated Human Carbonic Anhydrase Isoforms IX and XII. Int. J. Mol. Sci..

[25] Peerzada M.N., Khan P., Ahmad K. (2018). Synthesis, characterization and biological evaluation of tertiary sulfonamide derivatives of pyridyl-indole based heteroaryl chalcone as potential carbonic anhydrase IX inhibitors and anticancer agents. Eur. J. Med. Chem..

[26] Eldehna W.M., Nocentini A., Hassan G.S., Supuran C.T. (2018). Tumor-associated carbonic anhydrase isoform IX and XII inhibitory properties of certain isatin-bearing sulfonamides endowed with in-vitro antitumor activity towards colon cancer. Bioorg. Med. Chem..

[27] Mojzych M., Ceruso M., Supuran (2015). C.T. New pyrazolo[4,3e][1.2.4]triazino sulfonamides as carbonic anhydrase inhibitors. Bioorg. Med. Chem. Lett..

[28] Matysiak J., Skrzypek A., Tarasiuk P. (2017). QSAR study of pyrazolo[4,3e][1,2,4]triazino sulfonamide against tumor-associated human carbonic anhydrase isoforms IX and XII. Comp. Boil. Chem..

[29] Nocentini A., Ferraroni M., Carta F., Supuran C.T. (2016). Benzene sulfonamides incorporating flexible Triazole Moieties Are Highly Effective Carbonic anhydrase Inhibitors: Synthesis and Kinetic, Crystallographic, Computational and Intraocular Pressure Lowering Investigations. J. Med. Chem..

[30] Huy X.N., Green K.D., Gajadeera C.S. (2018). Potent 1,2,4-Triazino[5,6b]indole-3-thioether inhibitors of Kanamycin Resistance Enzyme Eis from Mycobacterium tuberculosis. ACS Infect. Dis..

[31] Patel D.V., Patel N.R., Kanhed A.M. (2019). Novel Multitarget Directed Triazinoindole Derivatives as Anti-Alzheimer Agents. ACS. Chem. Neurosci..

[32] Khalifah R.G. (1971). The carbon dioxide hydration activity of carbonic anhydrase. I. Stopflowkinetic studies on the native human isoenzymes B and C. J. Biol. Chem..

[33] Supuran C.T., Barboiu M., Luca C., Pop E., Brewster M.E., Dinculescu A. (1996). Carbonic anhydrase activators. Part 14. Synthesis of mono- and bis- pyridinium salt derivatives of 2-amino-5-(2-aminoethyl)- and 2-amino-5-(3-aminopropyl)-1,3,4-thiadiazole, and their interaction with isozyme II. Eur. J. Med. Chem..

[34] Öztürk Sarıkaya S.B., Gülçin I., Supuran C.T. (2010). Carbonic anhydrase inhibitors. Inhibition of human erythrocyte isozymes I and II with a series of phenolic acids Chem. Biol. Drug Des..

[35] Carta F., Aggarwal M., Maresca A., Scozzafava A., McKenna R., Supuran C.T. (2012). Dithiocarbamates: A new class of carbonic anhydrase inhibitors. Crystallographic and kinetic investigations. Chem Commun..

[36] Boztaş M., Çetinkaya Y., Topal M., Gülçin İ., Menzek A., Şahin E., Tanc M., Supuran C.T. (2015). Synthesis and carbonic anhydrase isoenzymes I, II, IX, and XII inhibitory effects of dimethoxybromophenol derivatives incorporating cyclopropane moieties. J. Med. Chem..

[37] De Simone G., Supuran C.T. (2012). (In)organic anions as carbonic anhydrase inhibitors. J. Inorg. Biochem..

[38] Innocenti A., Gülçin I., Scozzafava A., Supuran C.T. (2010). Carbonic anhydrase inhibitors. Antioxidant polyphenols effectively inhibit mammalian isoforms I–XV. Bioorg. Med. Chem. Lett..

