# Supplementary material for: Design, Synthesis, and Biological Evaluation of 1,2,3-Triazole-linked triazino[5,6-b]indole-benzene sulfonamide Conjugates as Potent Carbonic Anhydrase I, II, IX, and XIII Inhibitors
Source: Metabolites. 2020 May 15;10(5):200. doi: 10.3390/metabo10050200 (PMC7281265; doi:10.3390/metabo10050200)
Supplement: Supplementary file 1 [file metabolites-10-00200-s001.pdf]

Supporting information

Design, synthesis and biological evaluation of 1,2,3-triazole linked triazino[5,6-b]indole-benzene sulfonamide conjugates as potent carbonic anhydrase II, IX and XIII inhibitors

Krishna Kartheek Chinchilli[a], Andrea Angeli,[c] Pavitra S Thacker[a], Laxman Naik Korra[a], Rashmita Biswas[a], Mohammed Arifuddin[a,b]* and Claudiu T. Supuran[c]*

[a]Department of Medicinal Chemistry, National Institute of Pharmaceutical Education and Research (NIPER), Balanagar, Hyderabad 500037, India

[b]Department of Chemistry, Anwarul Uloom College, 11-3-918, New Malleypally, Hyderabad-500001, T. S., India (Present Address)

[c]Università degli Studi di Firenze, Neurofarba Dept., Sezione di Scienze Farmaceutiche e Nutraceutiche, Via Ugo Schiff 6, 50019 Sesto Fiorentino, Florence, Italy

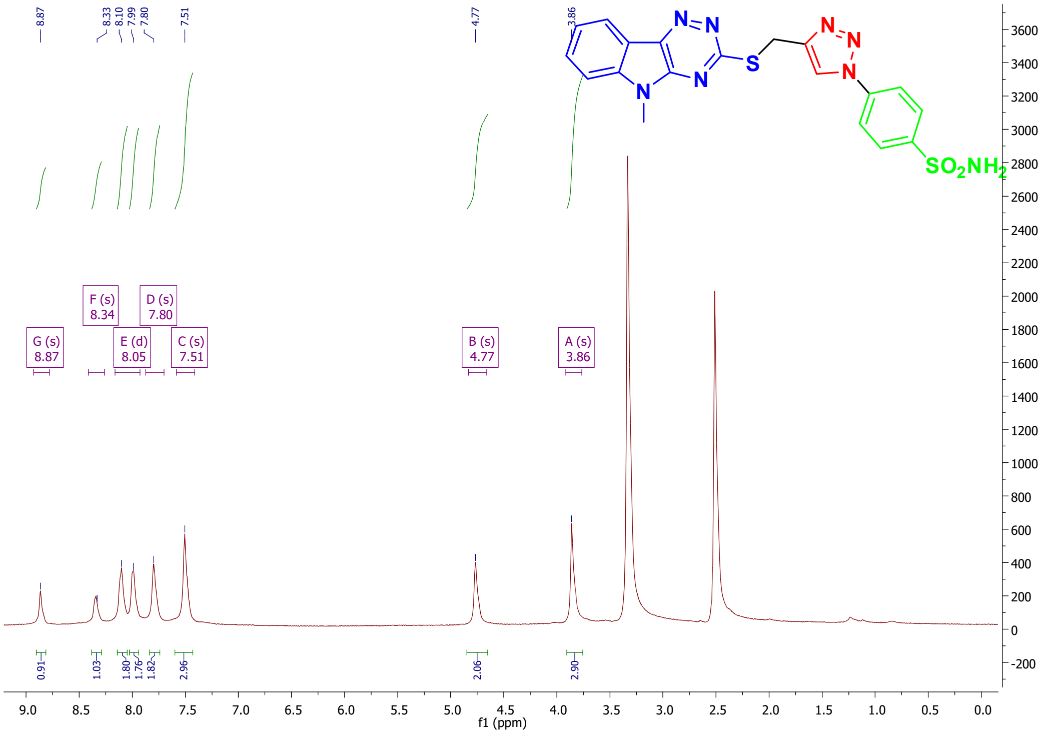

1H NMR spectra of 6a

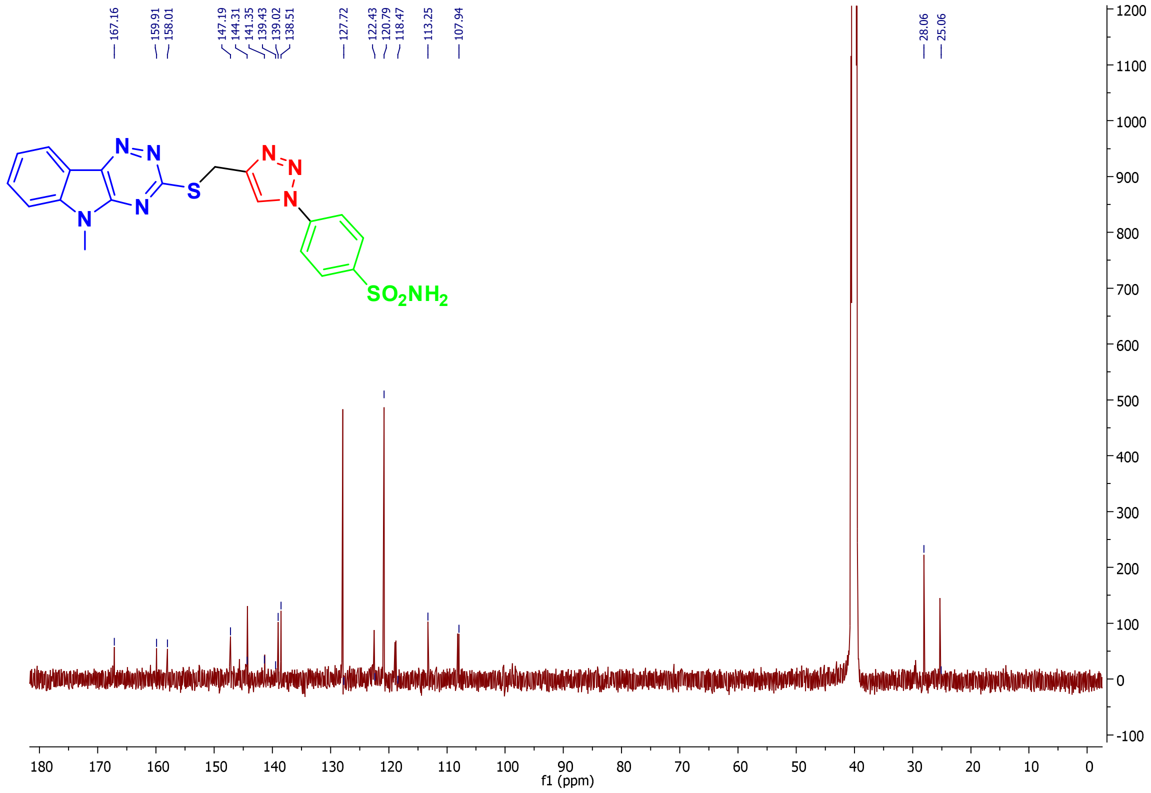

13C NMR spectra of 6a

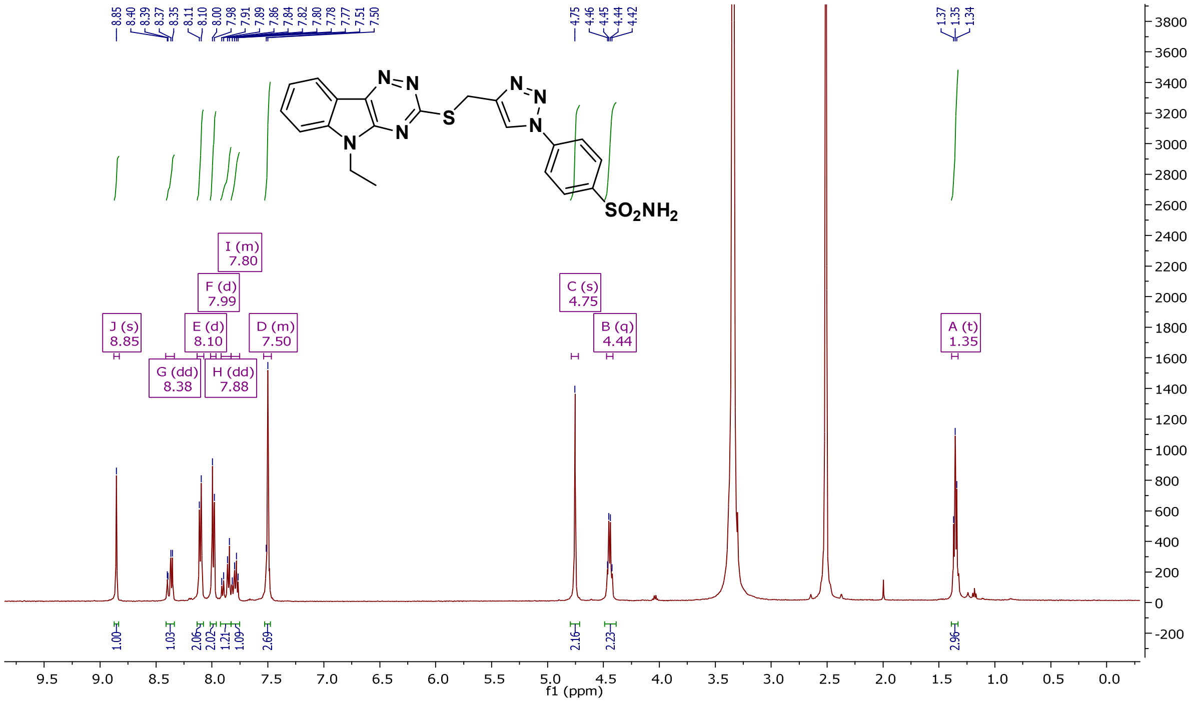

1H NMR spectra of 6b

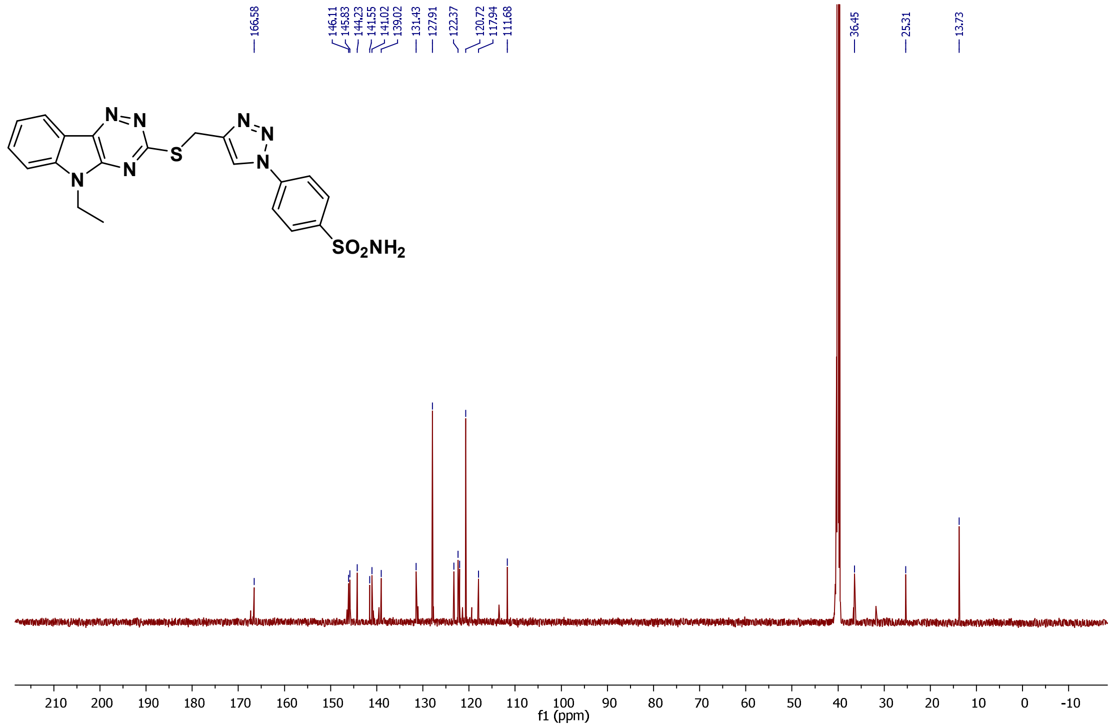

13C NMR spectra of 6b

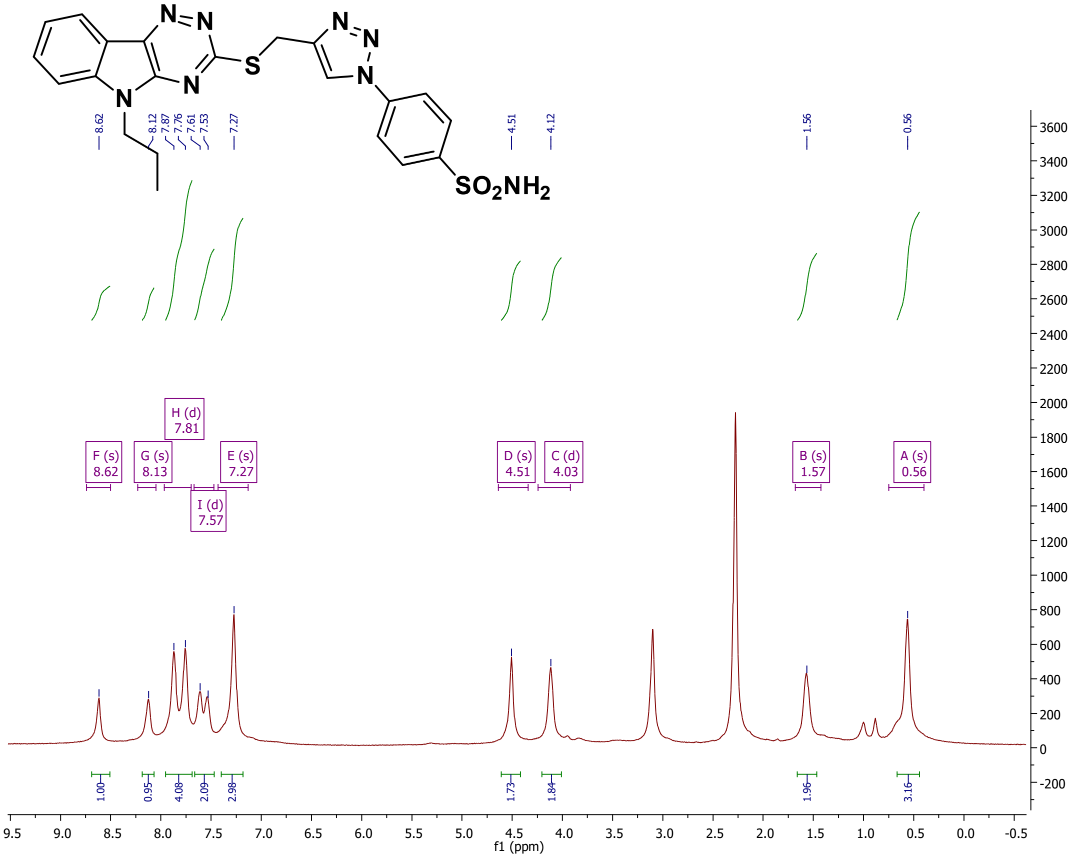

^{1}H NMR spectra of 6c

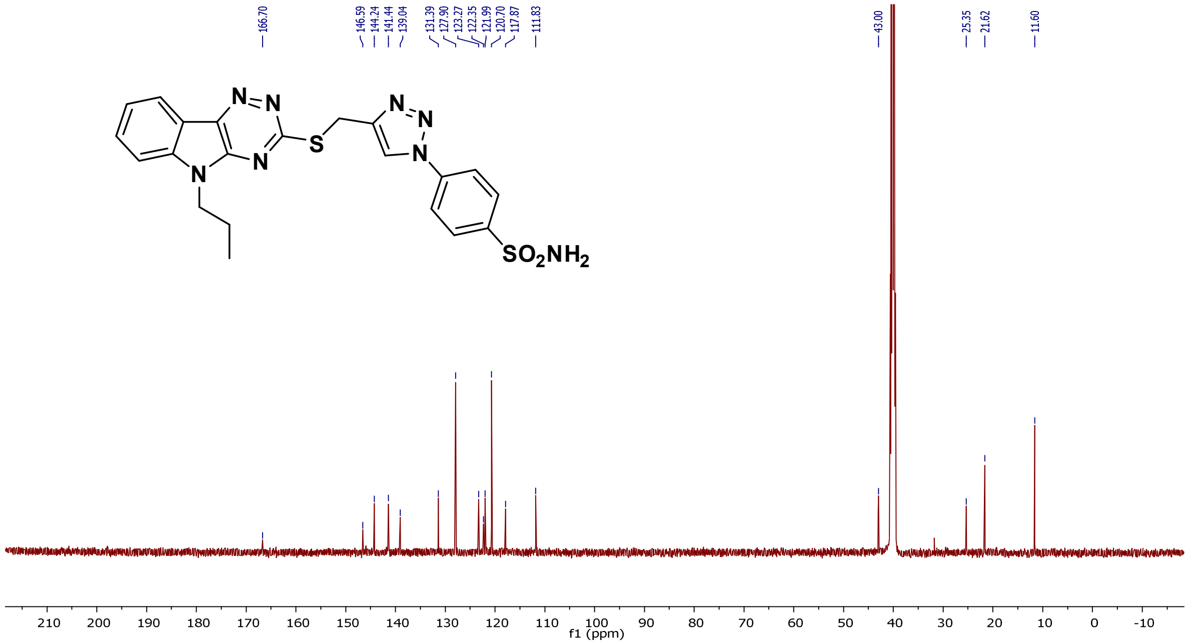

13C NMR spectra of 6c

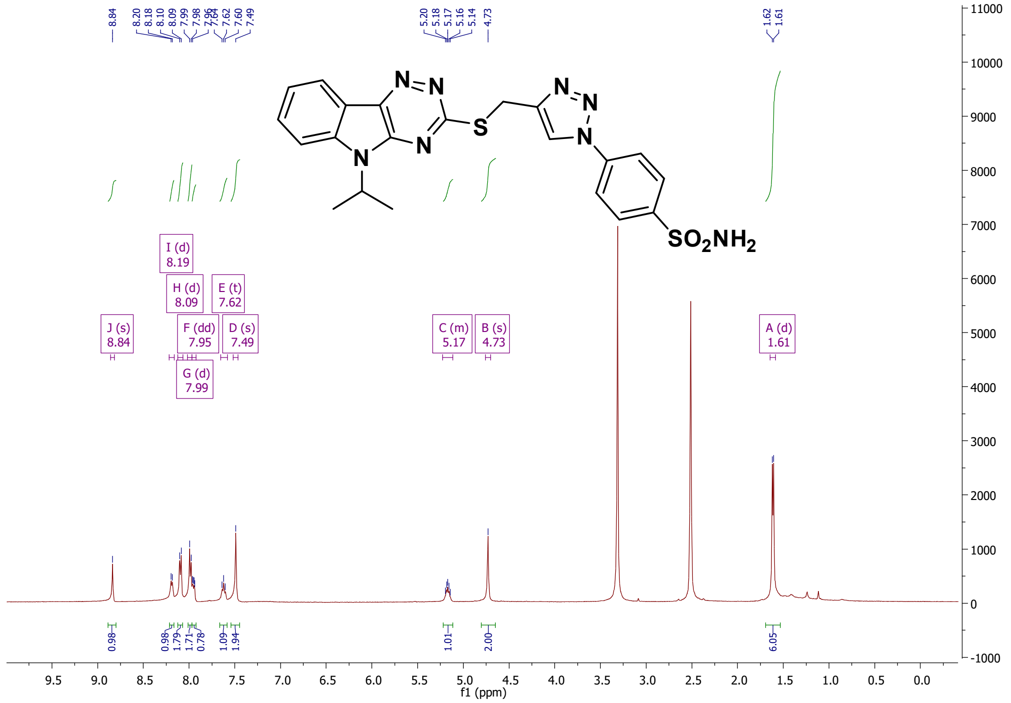

1H NMR spectra of 6d

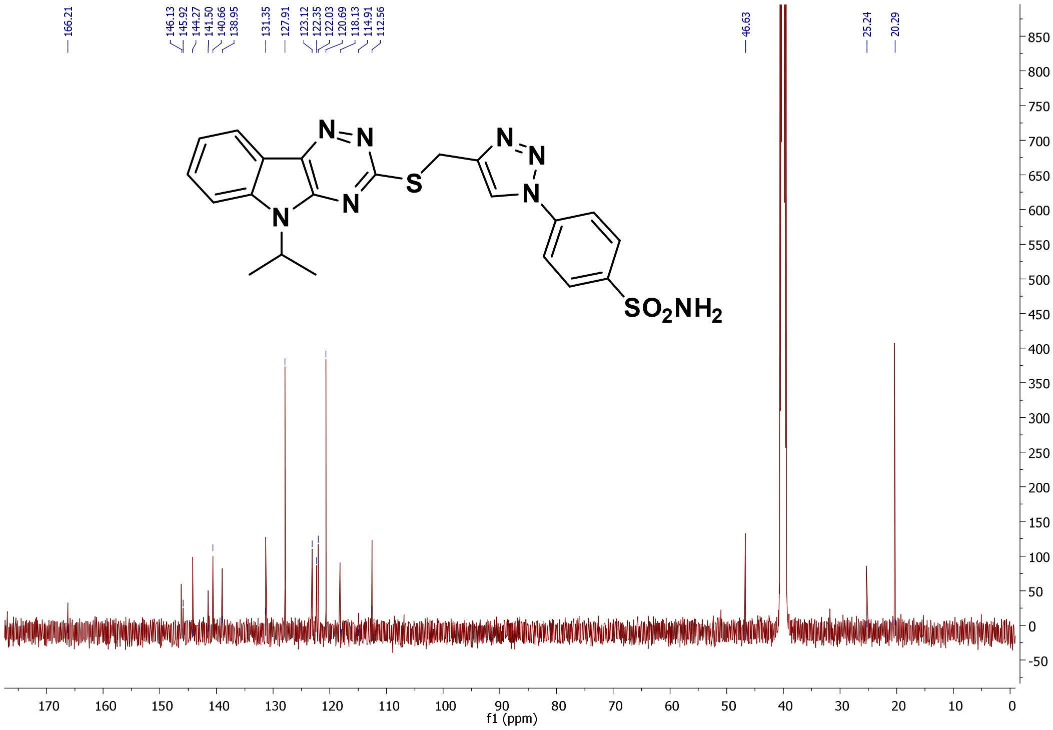

13C NMR spectra of 6d

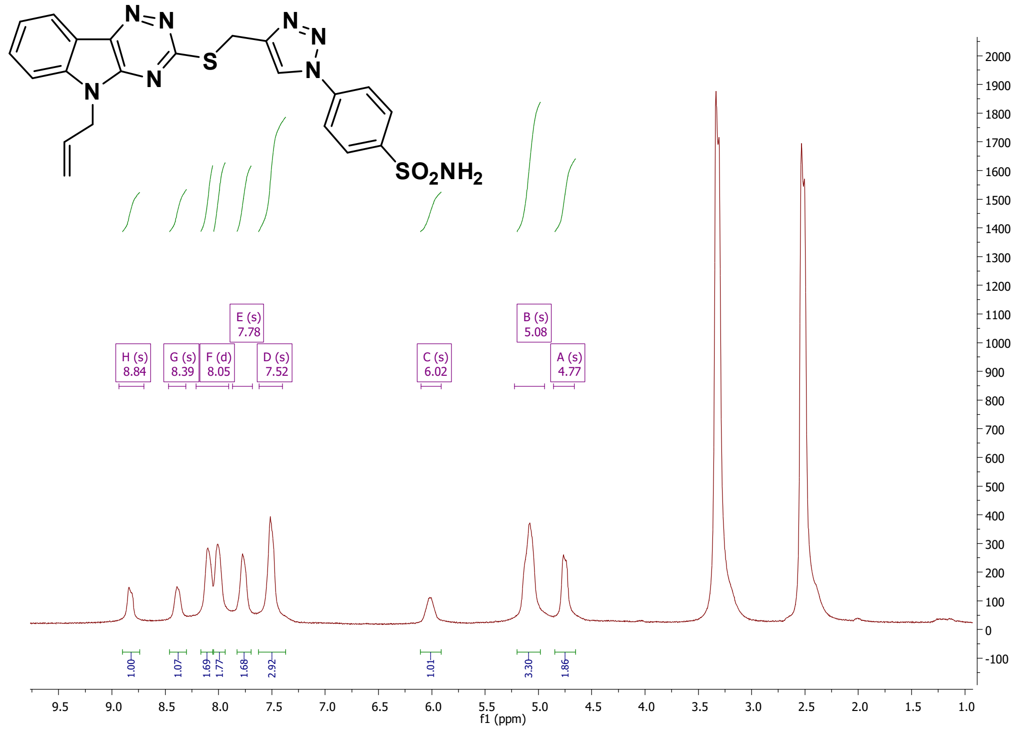

1H NMR spectra of 6e

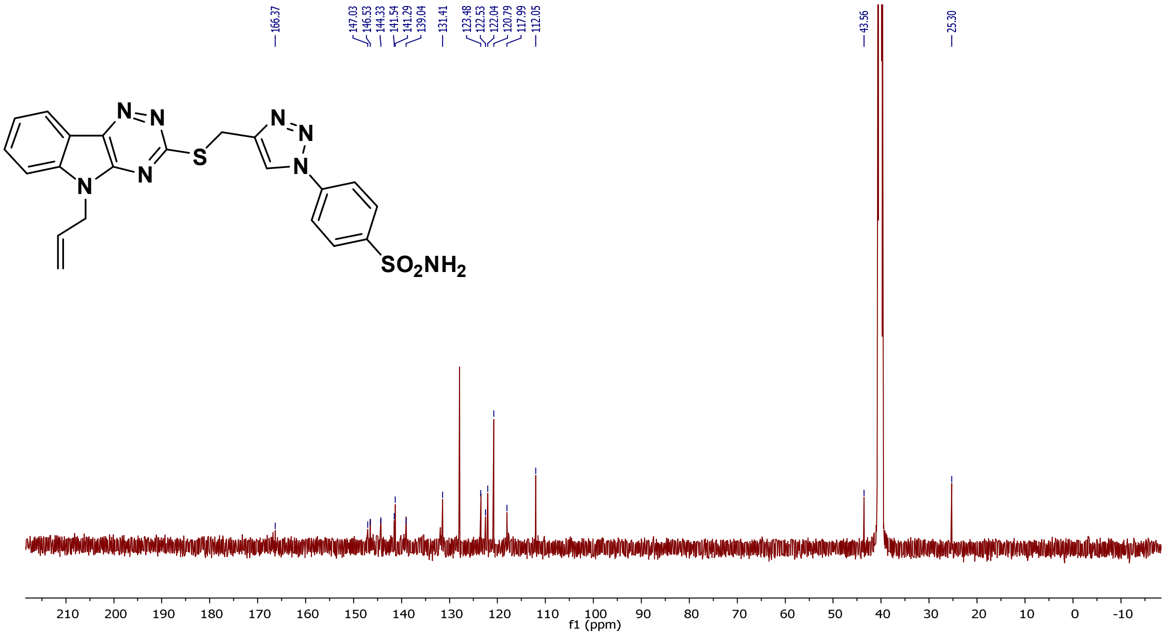

13C NMR spectra of 6e

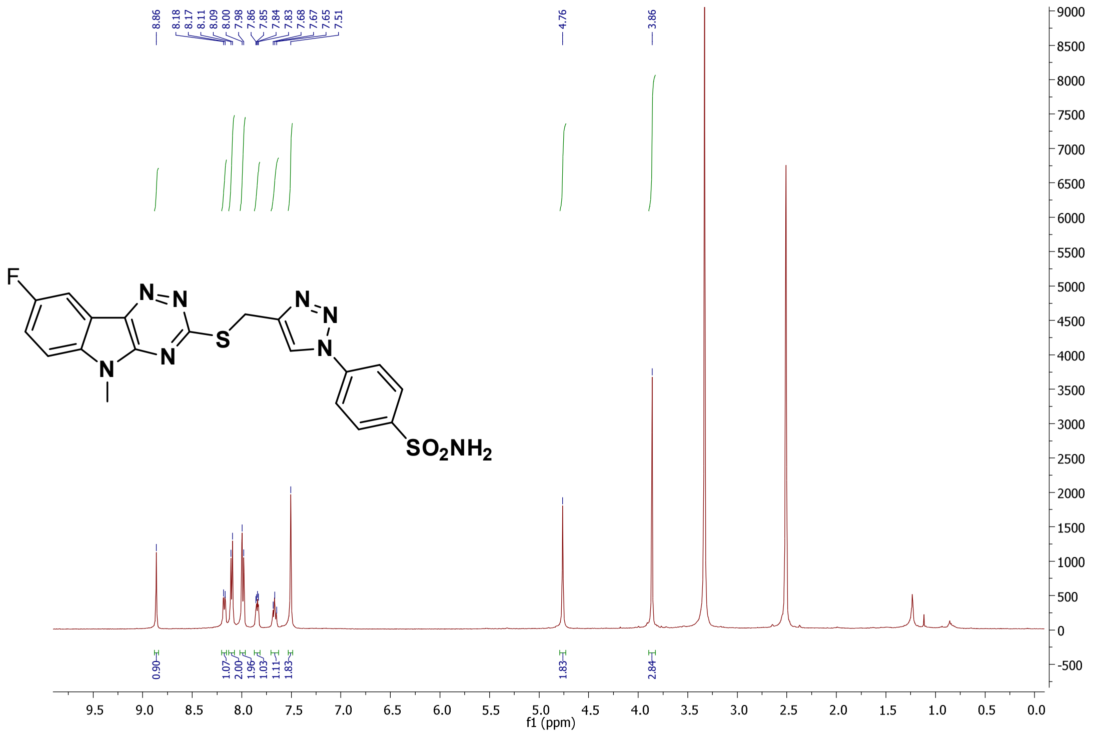

1H NMR spectra of 6f

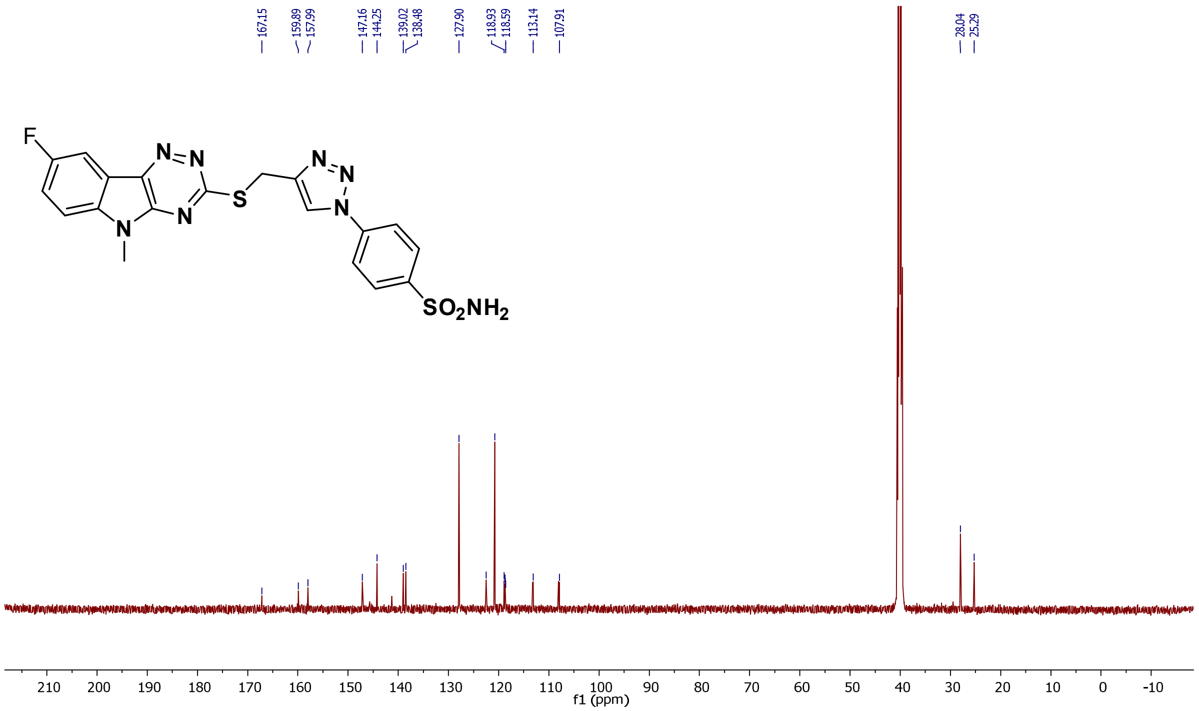

13C NMR spectra of 6f

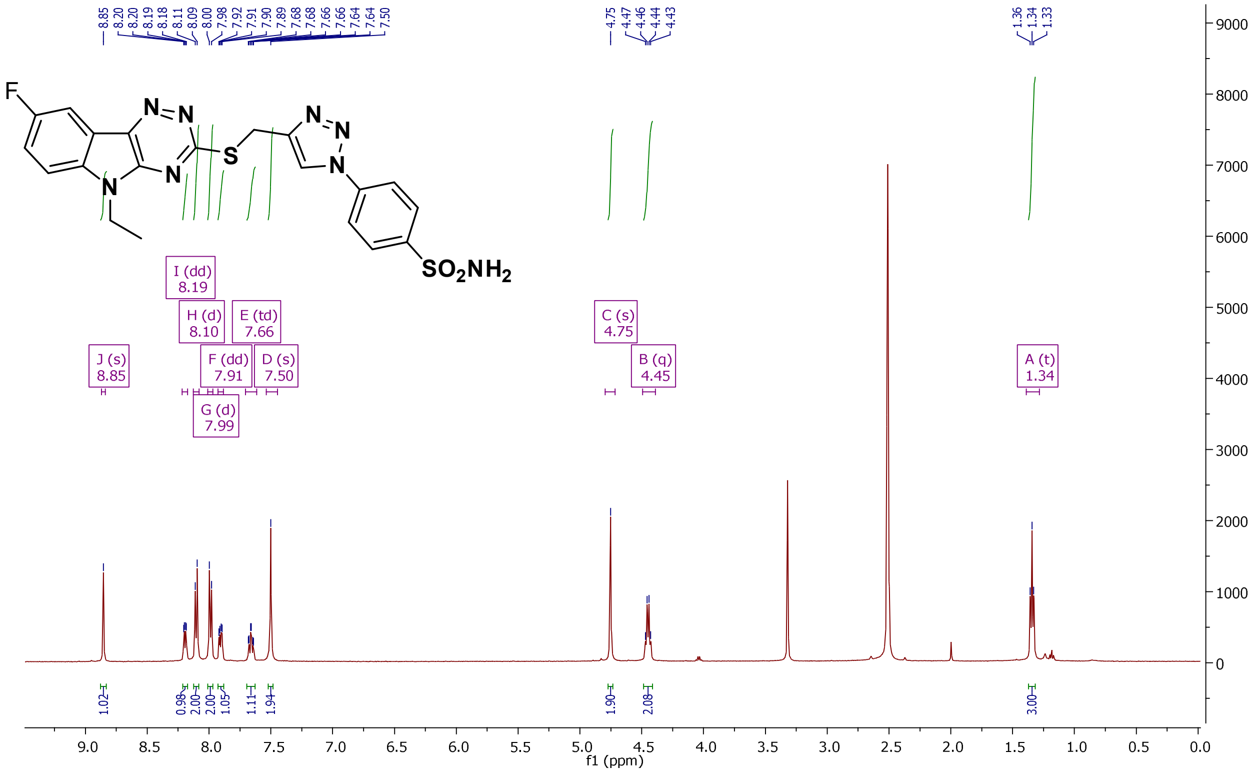

1H NMR spectra of 6g

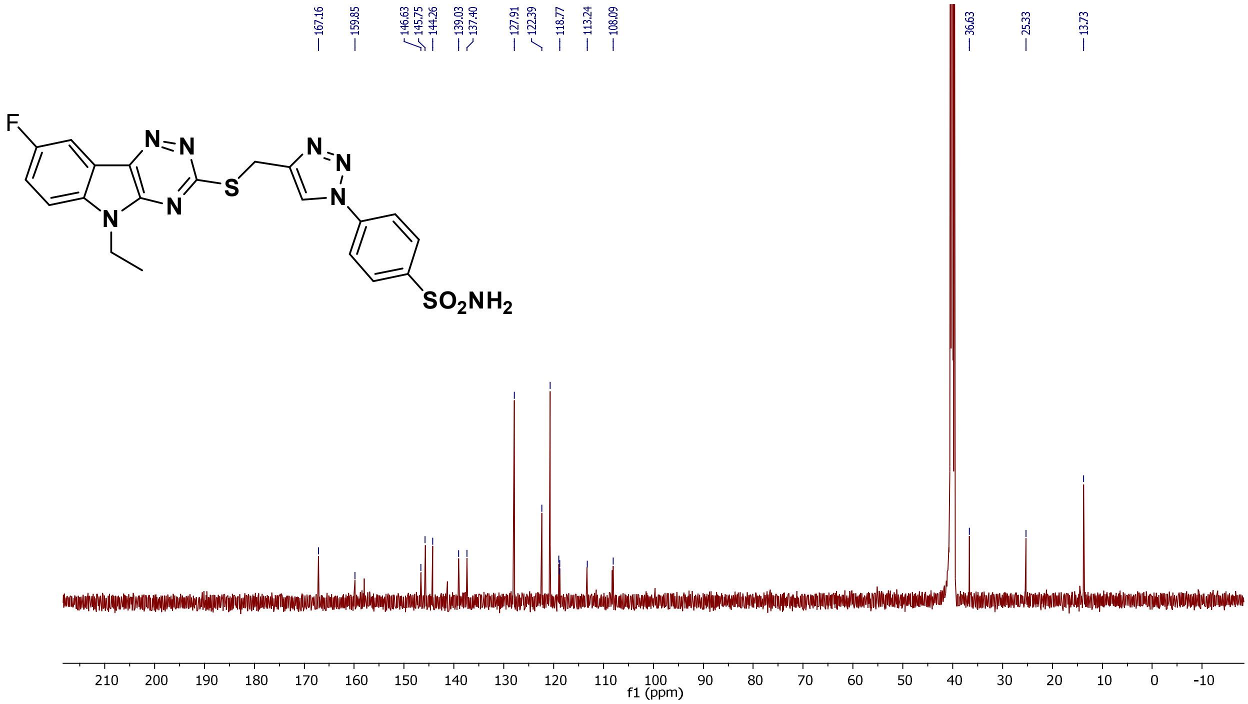

13C NMR spectra of 6g

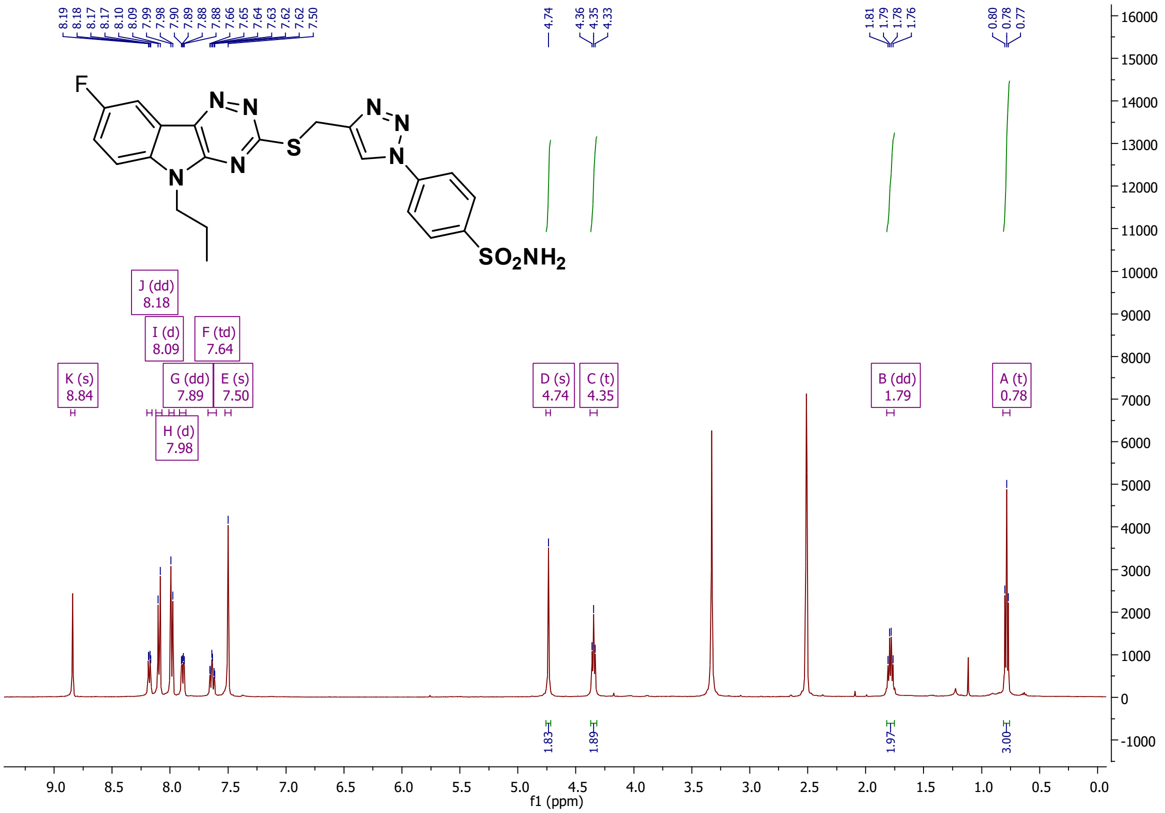

1H NMR spectra of 6h

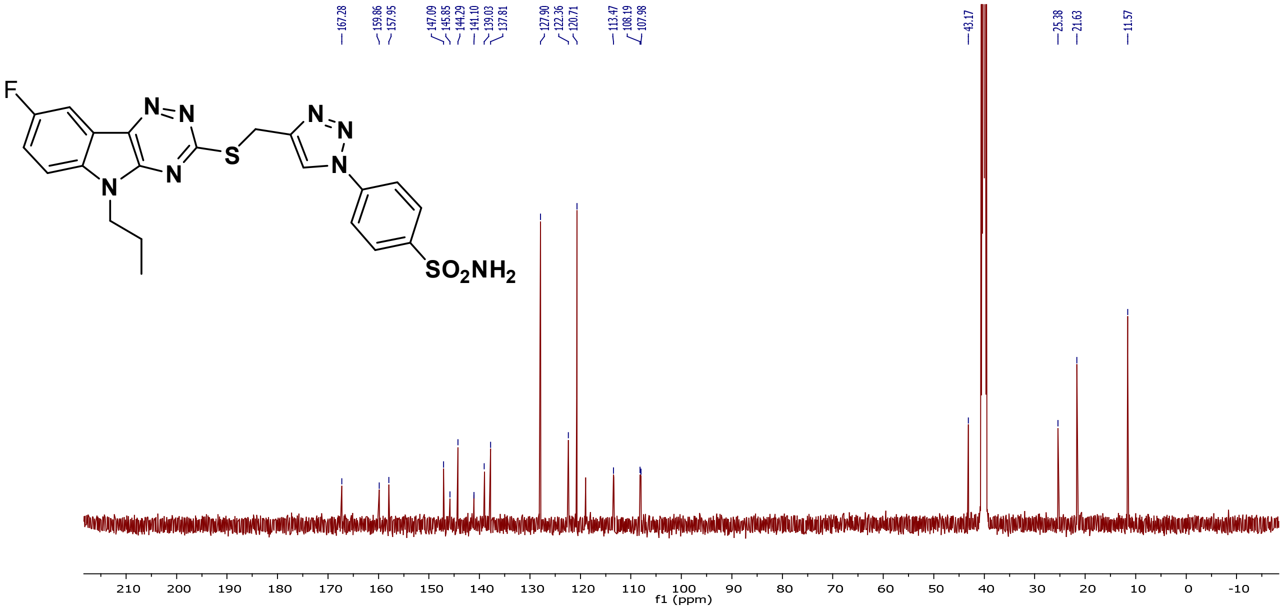

13C NMR spectra of 6h

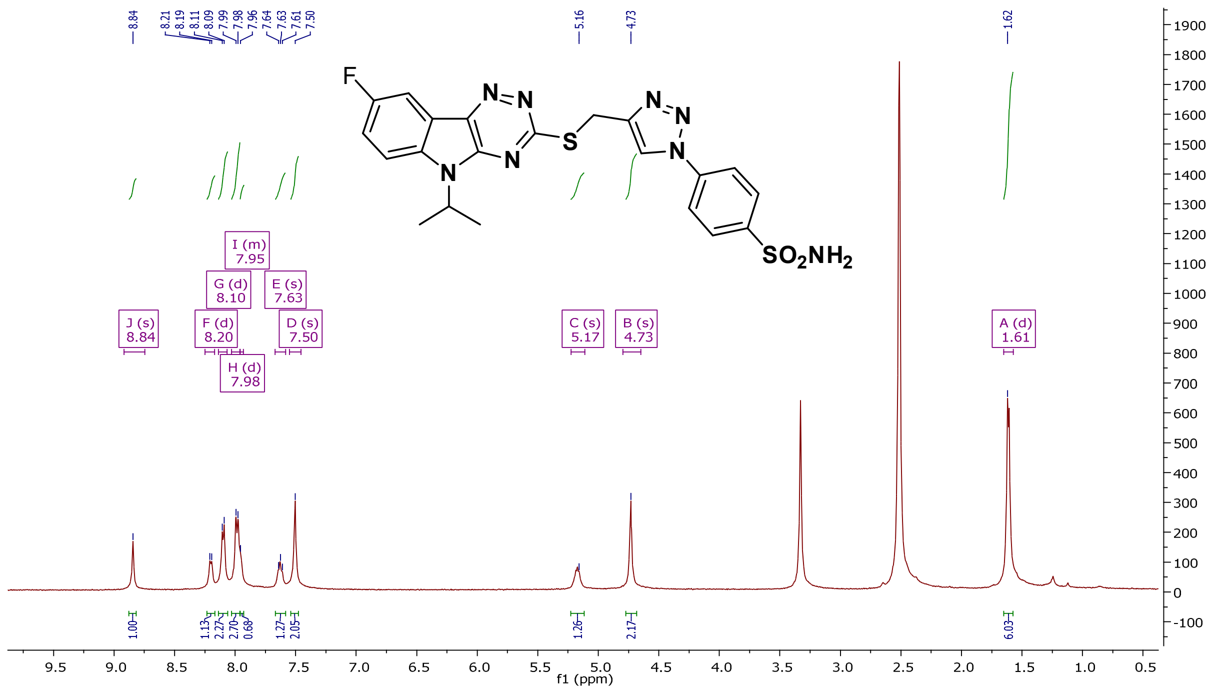

1H NMR spectra of 6i

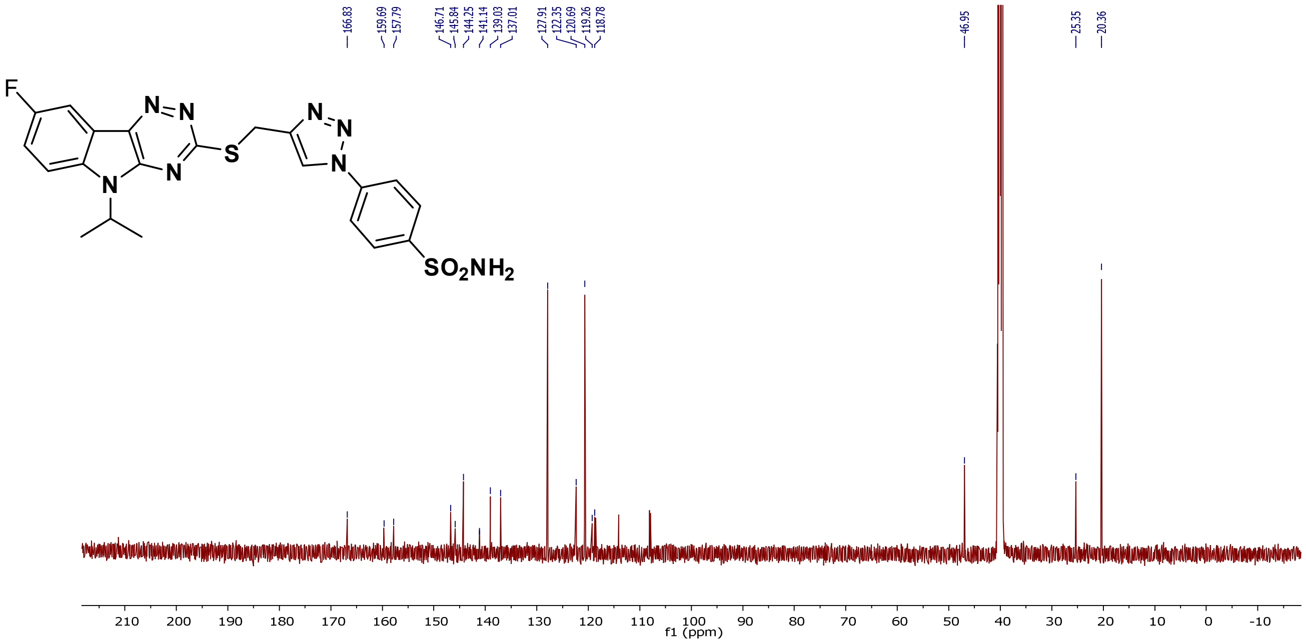

13C NMR spectra of 6i

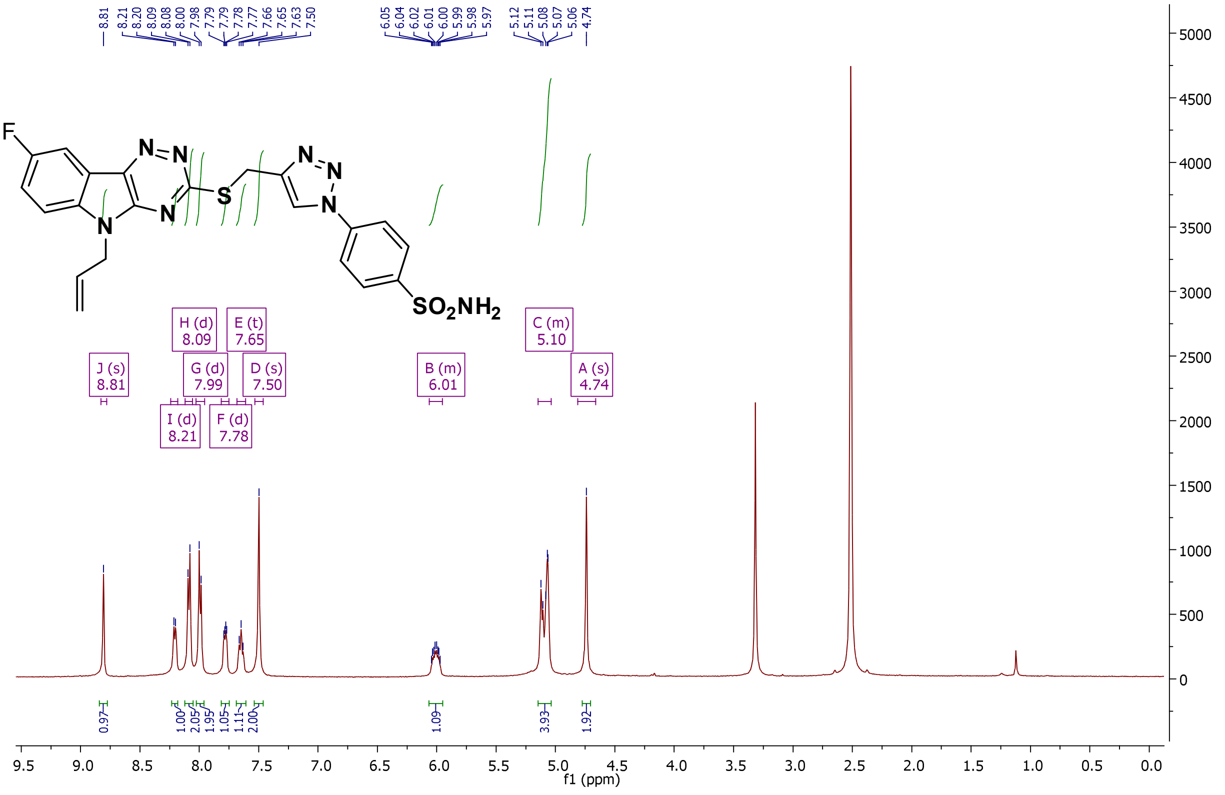

1H NMR spectra of 6j

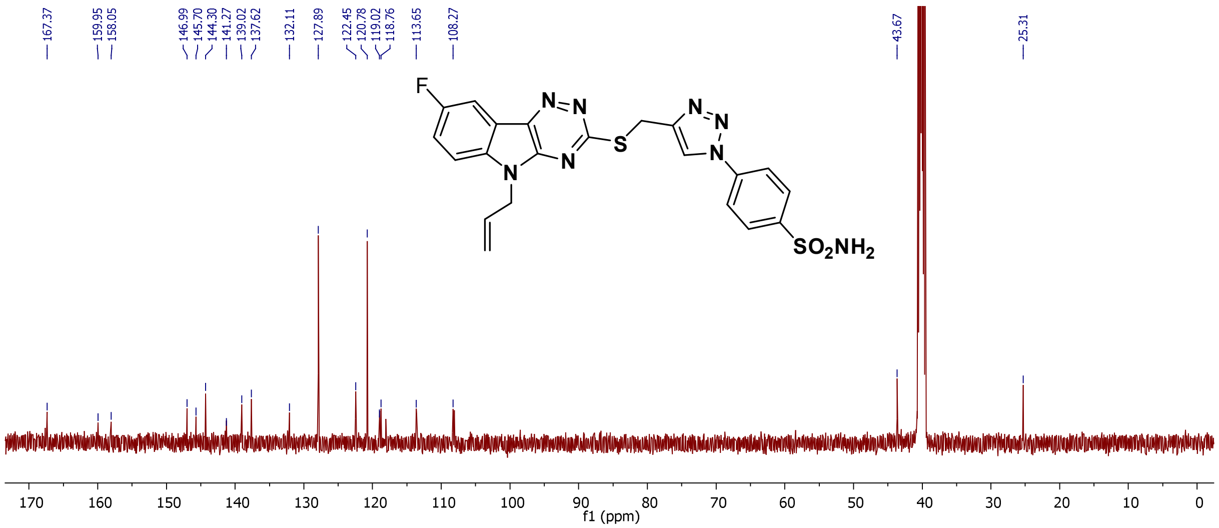

13C NMR spectra of 6j

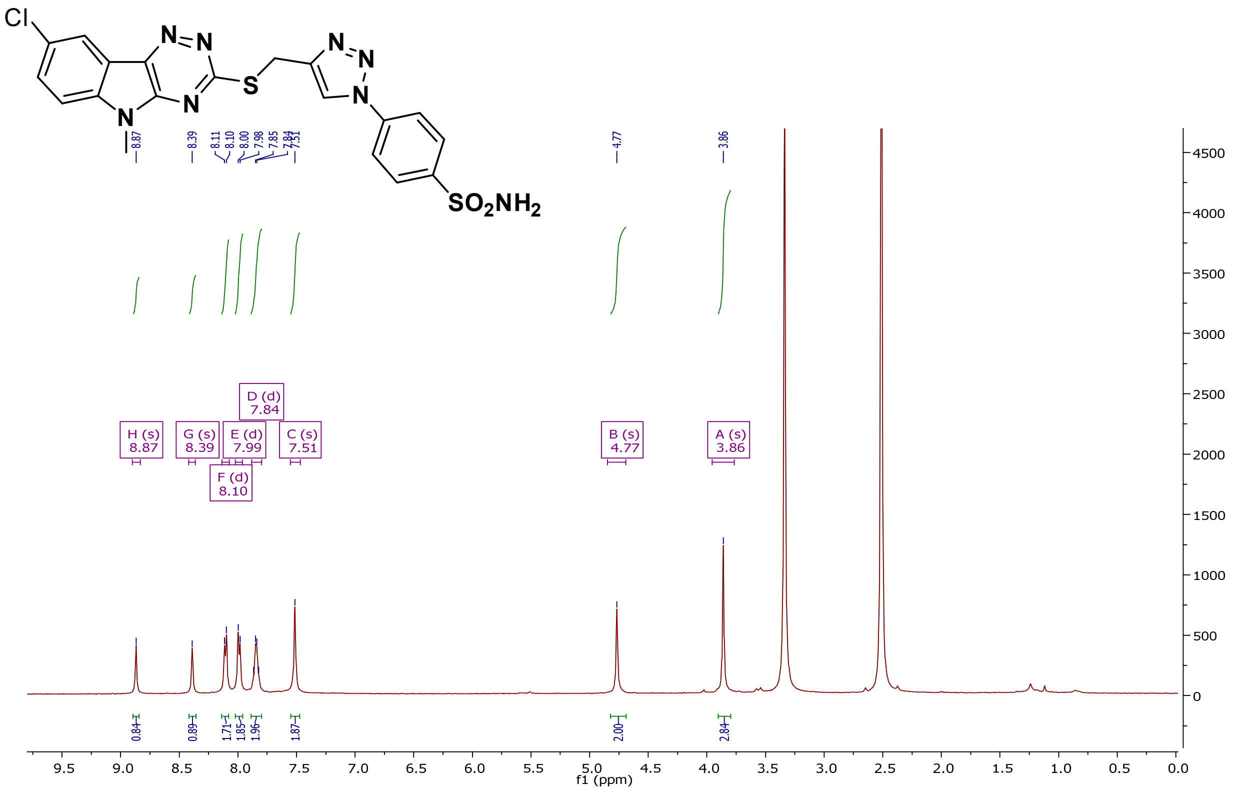

1H NMR spectra of 6k

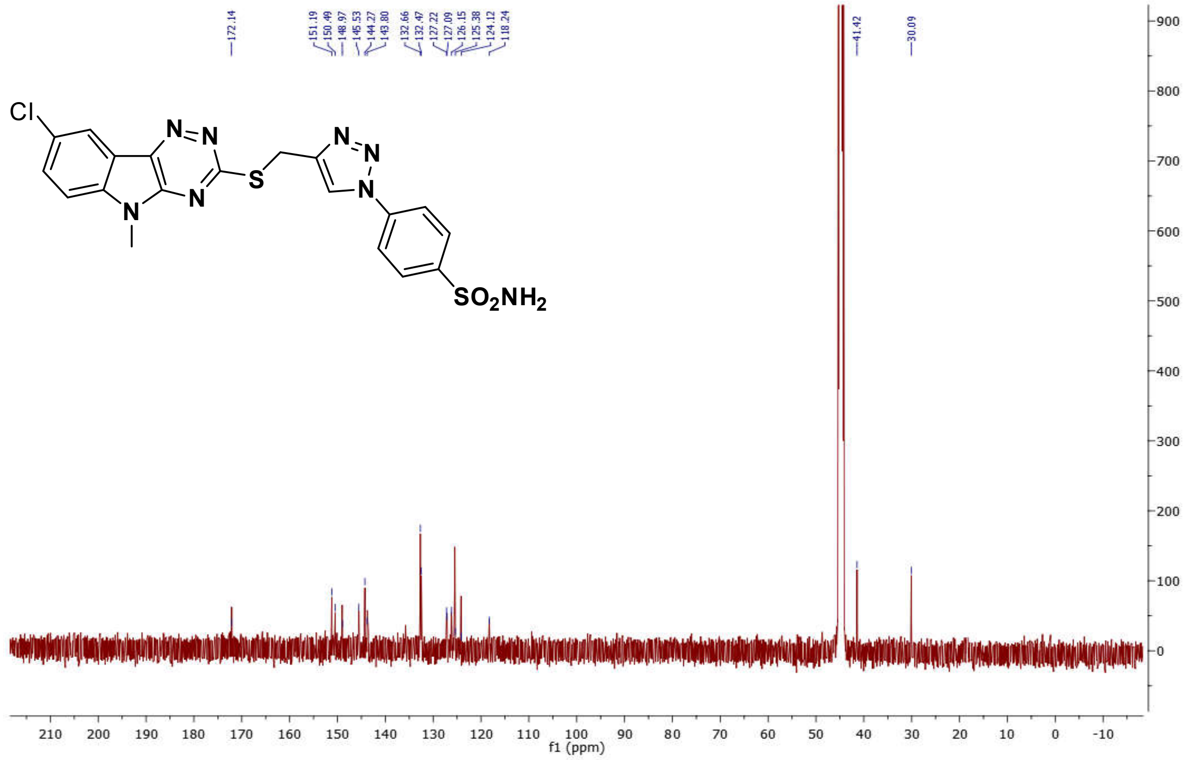

13C NMR spectra of 6k

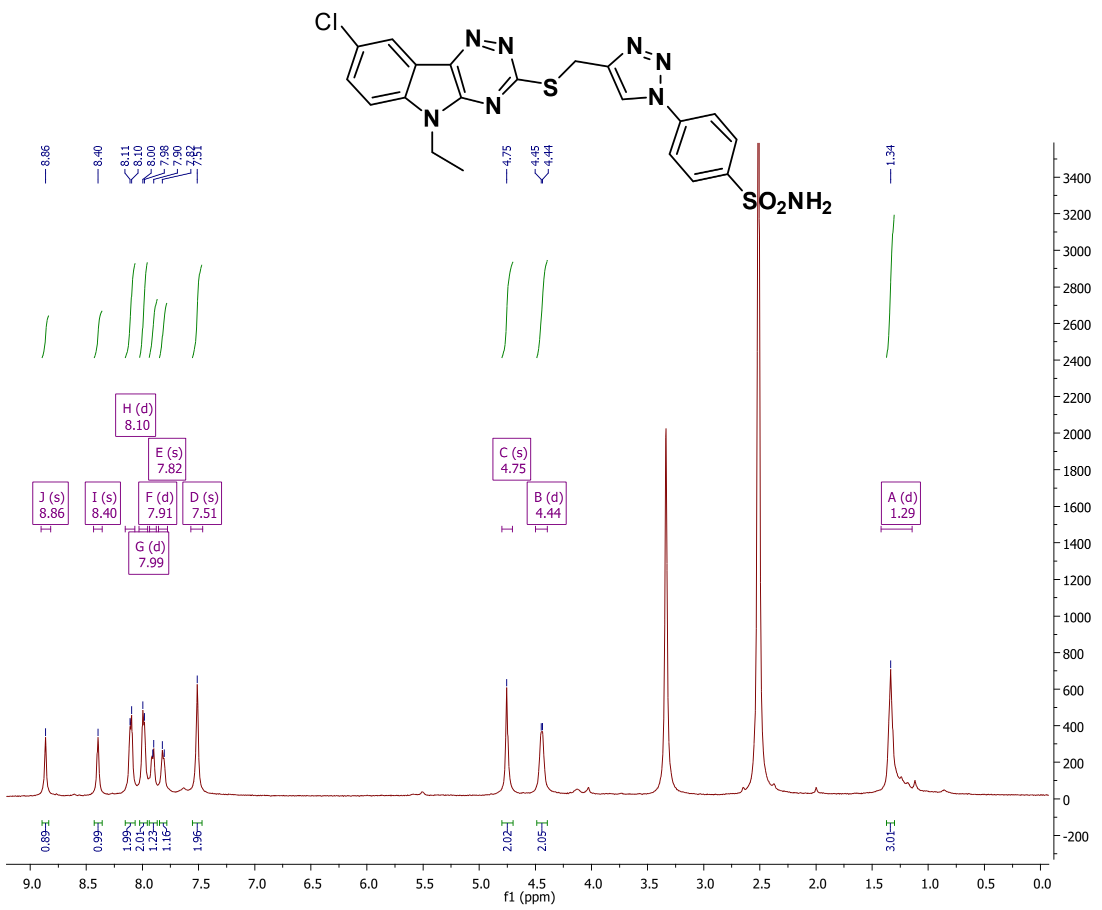

1H NMR spectra of 6l

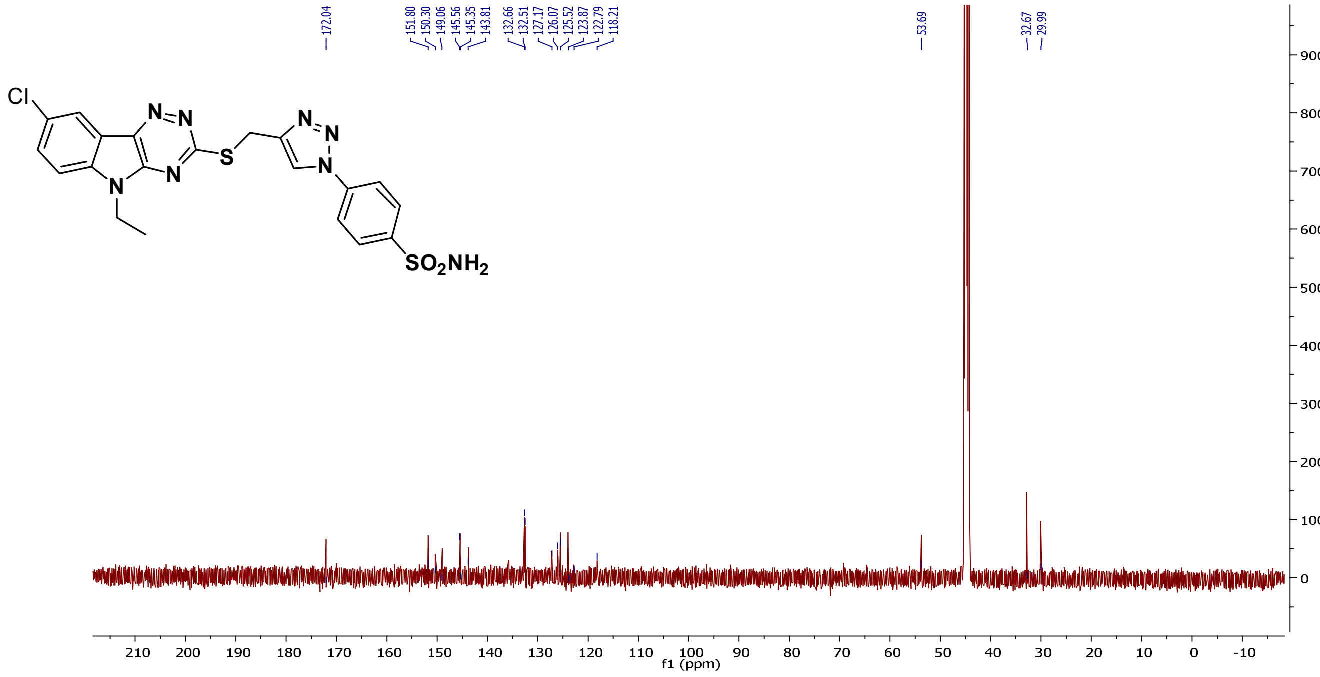

13C NMR spectra of 6l

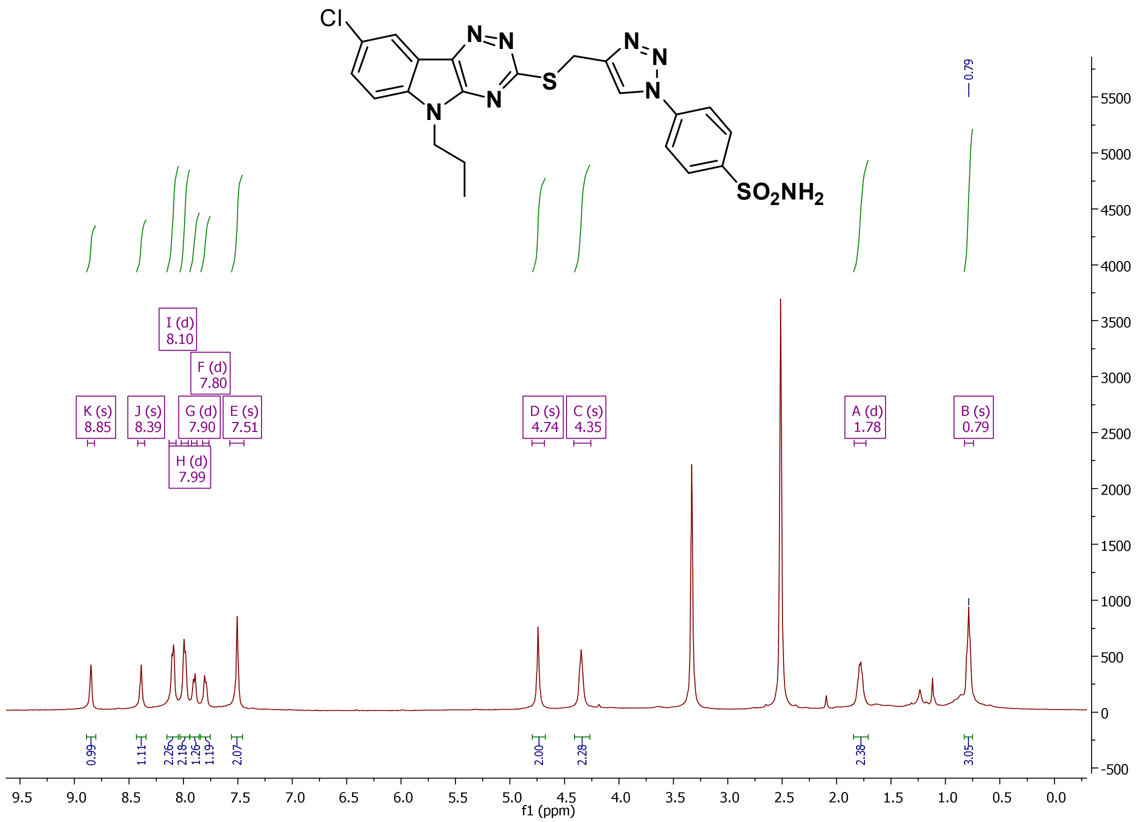

1H NMR spectra of 6m

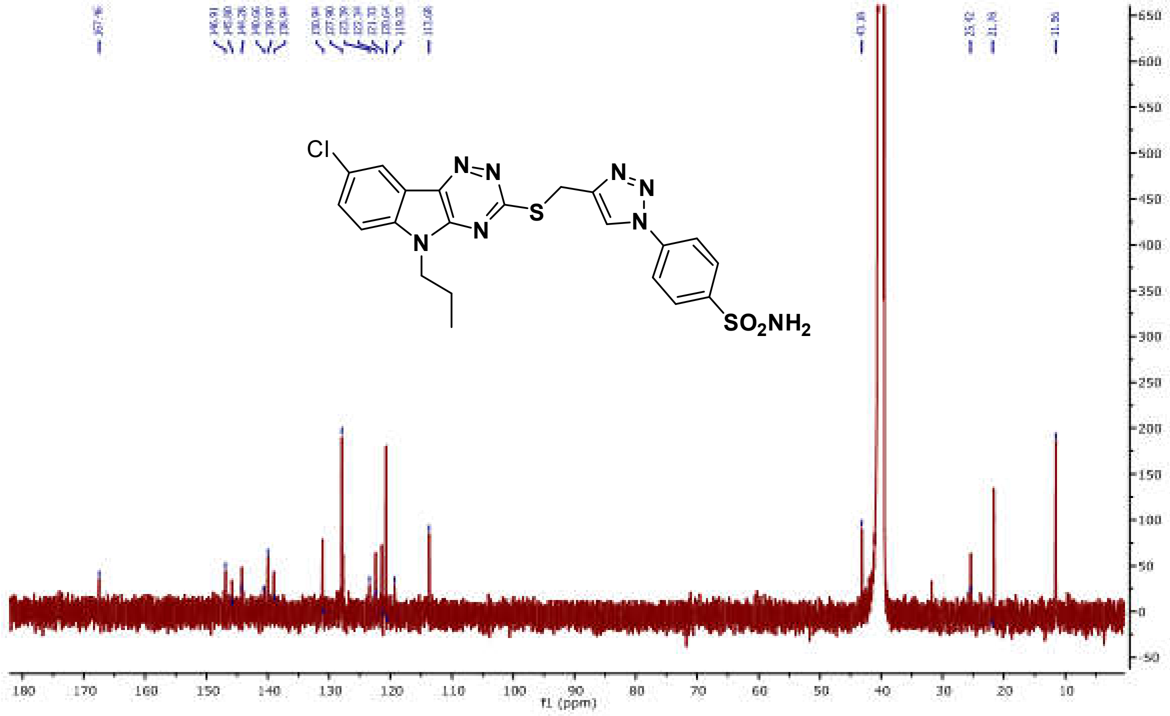

13C NMR spectra of 6m

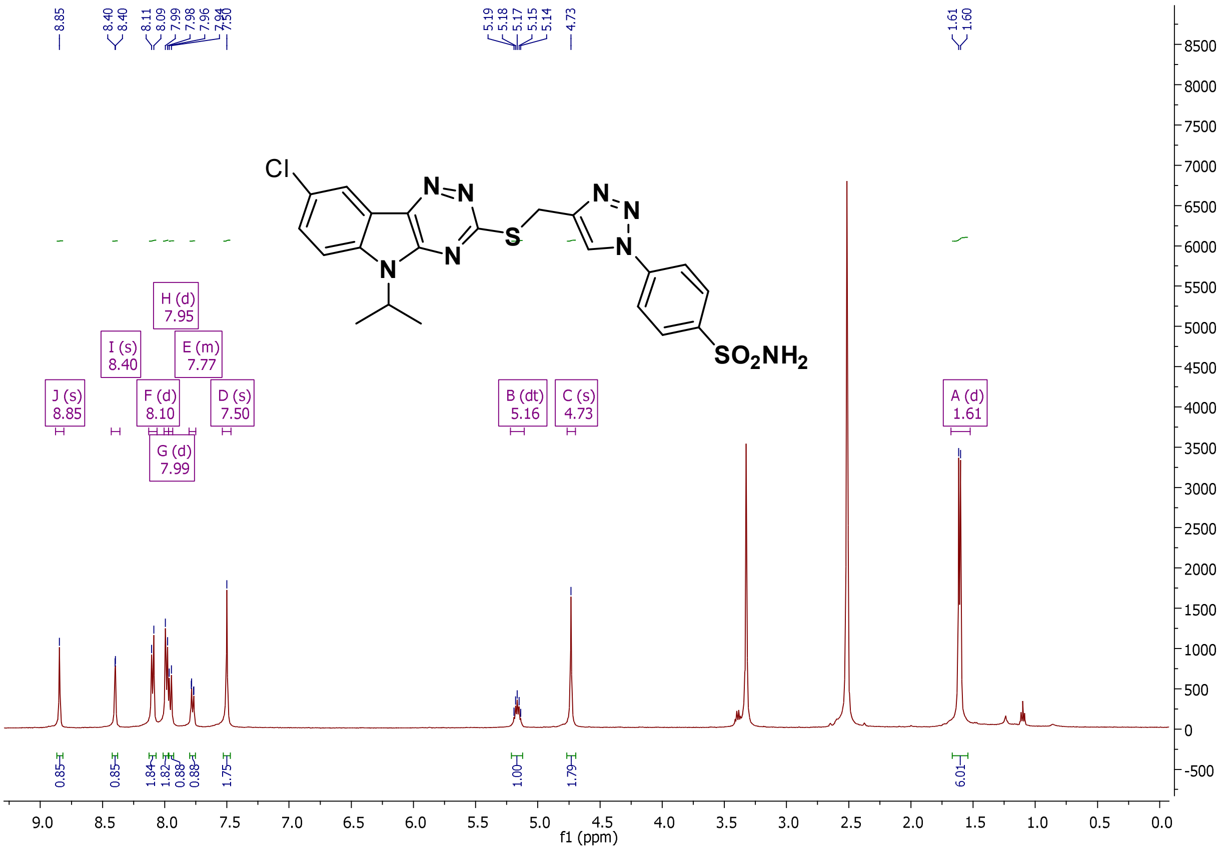

1H NMR spectra of 6n

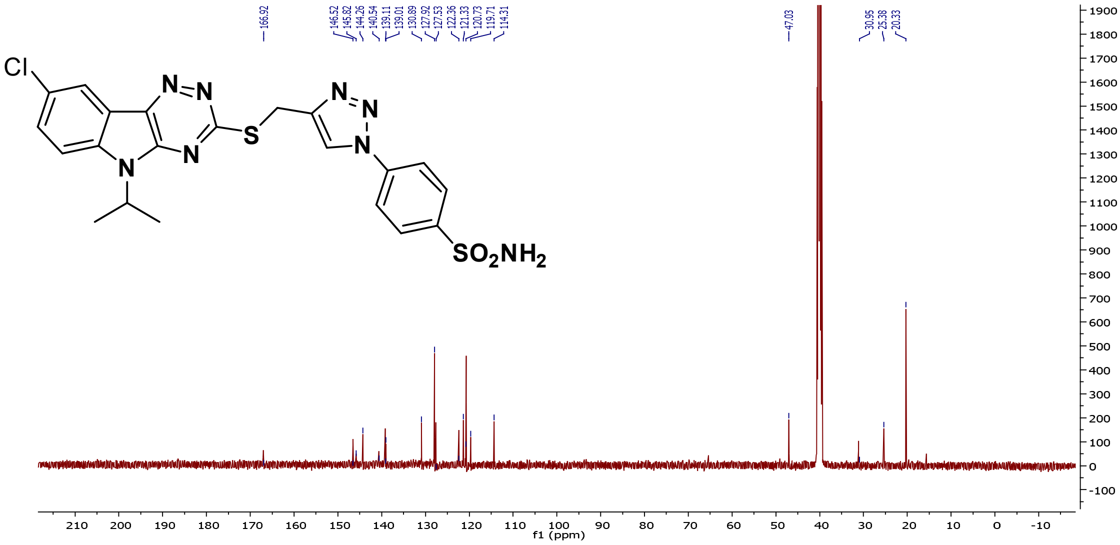

13C NMR spectra of 6n

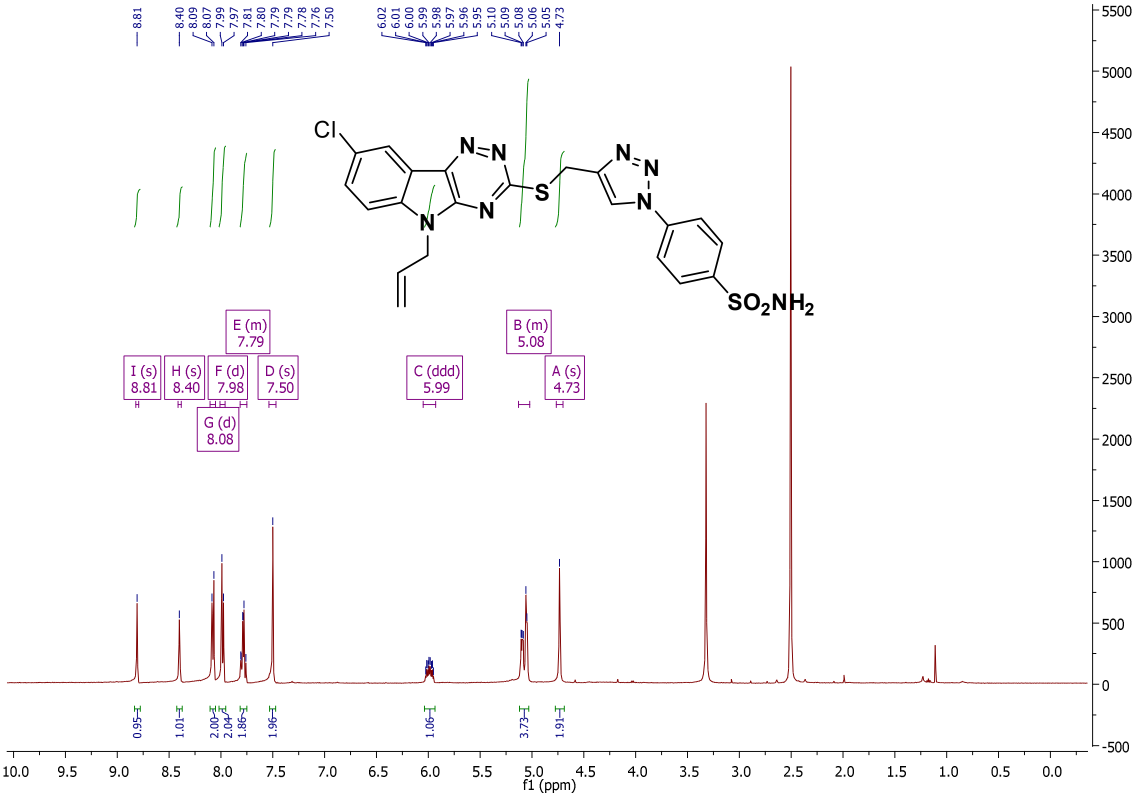

1H NMR spectra of 6o

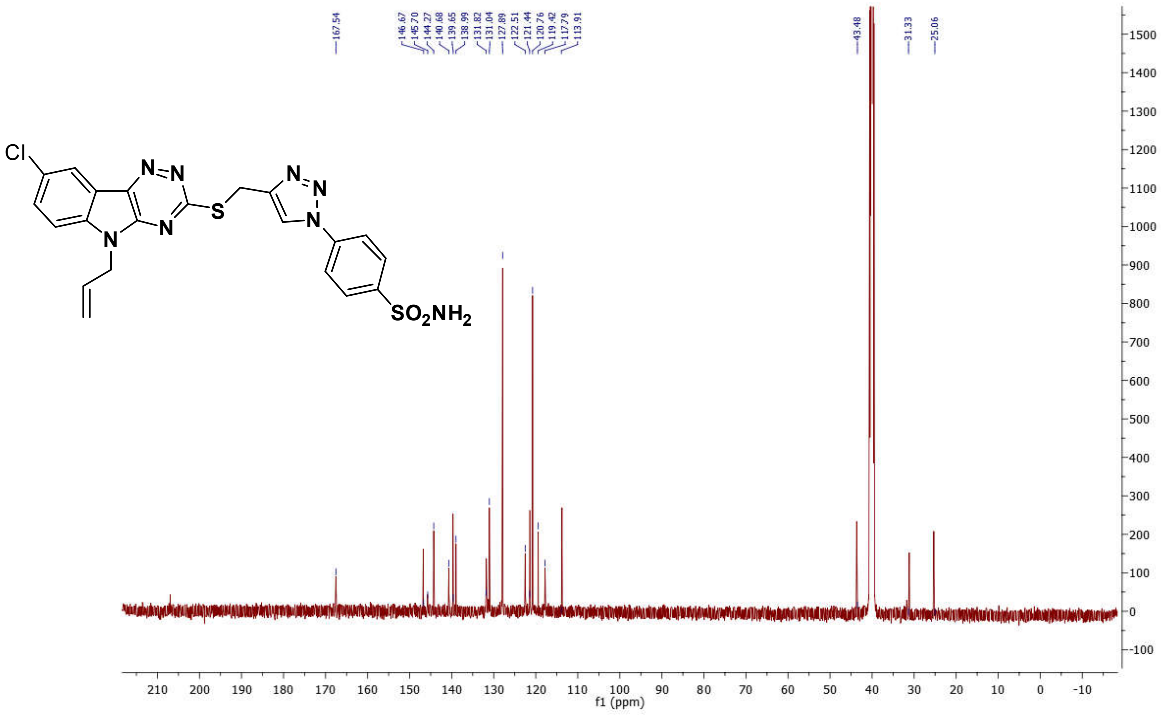

13C NMR spectra of 6o
